# From Dry to Wet, the Nature Inspired Strong Attachment Surfaces and Their Medical Applications

**DOI:** 10.1021/acsnano.4c17864

**Published:** 2025-03-07

**Authors:** Yurun Guo, Xiaobo Wang, Liwen Zhang, Xinzhao Zhou, Shutao Wang, Lei Jiang, Huawei Chen

**Affiliations:** † School of Mechanical Engineering and Automation, Beihang University, Beijing 102206, China; ‡ Technical Institute of Physics and Chemistry, Chinese Academy of Sciences, Beijing 100190, China; § Beijing Advanced Innovation Centre for Biomedical Engineering, Beihang University, Beijing 102206, China

**Keywords:** biomimetic, strong adhesion, strong friction, attachment mechanisms, interfacial dynamic behaviors, bioinspired surfaces, precision medicine, wearable electronics

## Abstract

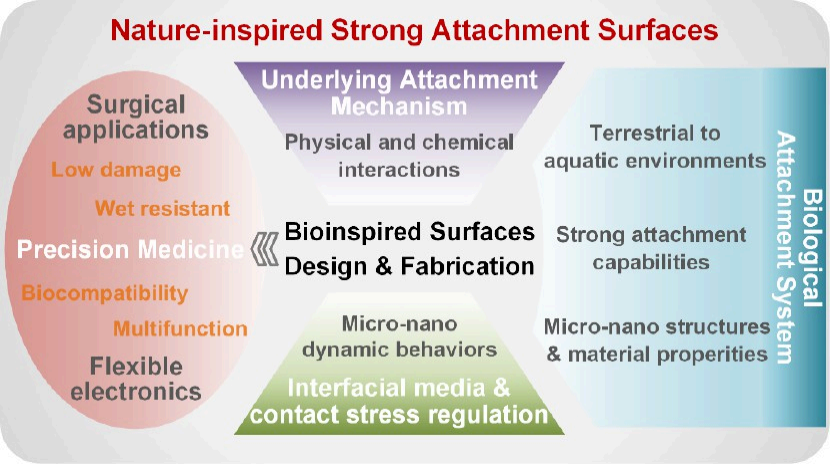

Strong attachment in complicated human body environments is of great importance for precision medicine especially with the rapid growth of minimal invasive surgery and flexible electronics. Natural organisms with highly evolved feet or claws can easily climb in complex environments from dry to wet and even underwater, providing significant inspiration for strong attachment research. This review summarizes the strong attachment behaviors of natural creatures in varied environments such as the gecko, tree frog, and octopus. Their attachment surfaces’ complex micronano structures and material properties exhibit evolutionary adaptations that enable them to transition across dry, wet, and underwater environments, highlighting the intricate mechanism of interfacial micronano dynamic behaviors. The interfacial liquid/air media regulation and contact stress adjustment from the coupling effects of surface structures and materials have been concluded as key factors in natural strong attachments. With the bioinspired strong attachment surface design, manufacturing methods including mold-assisted replication, nano 3D printing, self-assembly and field induced molding have been discussed. Finally, applications of bioinspired surfaces in low damage surgical instruments, tissue repair and flexible electronics have been demonstrated.

The demand for strong attachment in medical applications has become increasingly critical with the evolving requirements of precision medicine in areas such as minimally invasive surgical instruments and wearable electronics. As the most common mode of contact, the tissue/medical device biointerface requires robust and controllable attachment to ensure secure grasping or highly sensitive sensing functions.^1−4^ However, the human body presents a highly complex environment, characterized by air–liquid mixture, slippery mucus, multilevel surface roughness, and tissues with varying elastic properties, all of which pose significant challenges for achieving effective biointerface attachment. Due to the absence of micronano contact mechanisms, the design of functional surfaces for strong and controllable attachment still lacks a theoretical foundation. Therefore, innovative attachment solutions must provide strong, low-damage, and wet-resistant adhesion to ensure the reliability and effectiveness of medical devices. In nature, the ability to crawl or climb via strong adhesion or friction is essential for organisms to survive in complex environments. Over millions of years of evolution, diverse habitats with distinct conditions such as dry, wet, and even underwater, have led organisms to develop remarkable interfacial attachment strategies to effectively move and cling. These natural attachments can provide significant inspiration for developing advanced biomimetic surfaces for medical applications, enabling strong, reliable and adaptive attachment in complex and dynamic medical environments.

Based on the interfacial water volume, natural attachment strategies can be categorized into dry, wet, and underwater types, each presenting distinct demands and challenges.^5^ Typically, dry attachment requires effective adhesion and friction across surfaces ranging from smooth to highly rough, while wet attachment needs to overcome slippage caused by interfacial liquid films. In underwater conditions, the impacts of buoyancy and fluid dynamics make it difficult to generate the preload that are necessary for effective attachment. Compared to the most common method of contact with sharp stiff claws by creating mechanical interlocks, some natural creatures employ more advanced strategies by utilizing remarkable interfacial micronano effects to generate strong attachment in these dynamic environments. Representatively, the gecko with its pads can climb from nanosmooth glass to rough walls,^6^ tree frog with its mucus covered toe pads can freely climb on wet leaves,^7^ and the octopus with its active soft suckers can cling to various surfaces underwater.^8,9^ By exploring the underlying attachment mechanisms in different environments and mimicking their surface structures and materials, researchers have made significant progress in developing biomimetic adhesive surfaces.

On these creatures, various unique surface structures have been observed, such as long-thin nanofibers array, hierarchical micronano pillars array and diverse suckers.^10−13^ Their surfaces also exhibit particular material properties, including wettability,^14^ gradient stiffness,^15^ and special adhesive mucus.^16^ Various interfacial dynamic behaviors have been revealed such as liquid/air media regulating and the contact stress controlling at micronano scale, which have been ascribed to the formation of their strong contact. Boosted by these interfacial dynamic effects, the basic interfacial interaction such as van der Waals force,^17,18^ surface tension,^19^ capillary force,^7,20,21^ negative pressure,^8,22^ mechanical interlocking,^23−25^ and chemical bonding,^26^ have been adopted by creatures to create extraordinary strong attachment. Ranging from dry to wet conditions, these bioinspired strong attachment surfaces surpass conventional methods and offer significant advantages in medical applications, such as improved stability, minimal tissue damage, and enhanced reliability in diverse physiological environments.^11,27−32^ By systematically integrating principles from biological attachment strategies, it is possible to design medical adhesives that not only offer superior performance in strength and durability, but also possess the adaptability required for dynamic medical environments.^33,34^ The biomimetic approach to adhesion holds great promise for advancing the development of next-generation medical devices, enhancing their functionality, safety, and user comfort. The effects of interfacial structures and materials coupling on attachment mechanisms should be comprehensively summarized. These effects span diverse environments, ranging from dry to wet conditions, and encompass structural features across multiple scales—from micro- and nanoscale to the molecular level.

This review summarizes the typical natural strong attachment surfaces across dry, wet, and underwater environments (Figure 1). The evolutionary strategies of these creatures for adapting to different conditions have been compared in progressive changes of micronano structure and material properties. Their underlying strong attachment mechanisms have been categorized into micronano interfacial liquid/air media regulation and interfacial stress distribution adjustment. The design and fabrication of bioinspired surfaces for diverse environments are discussed, along with the applications of strong attachment surfaces in medical including surgical instruments, tissue repair and flexible electronics. Finally, this review presents the challenges in the design and manufacturing of biomimetic adhesive surfaces and explores potential future development trends.

**Figure 1:**
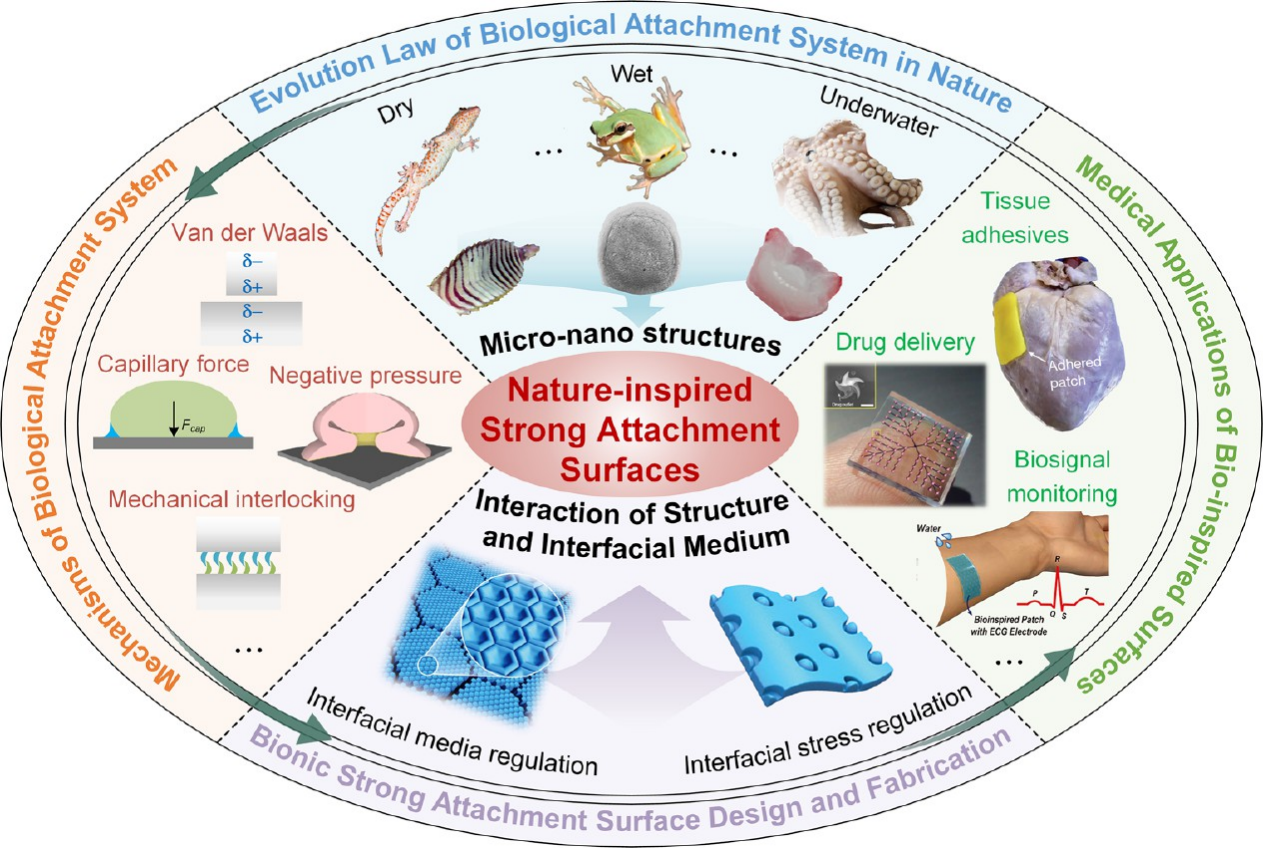
Schematic diagram illustrating the review of nature-inspired strong attachment surfaces. Nature has evolved a variety of organisms with exceptional adhesive properties, adapted to diverse humidity conditions ranging from dry to underwater environments. Examples include geckos, tree frogs, and octopuses, which display an evolutionary pattern in biological attachment systems. These organisms rely on delicate micro and nanostructures and materials to achieve robust adhesion through various attachment mechanisms, providing inspiration for the design and fabrication of artificial attachment surfaces. Bioinspired surfaces leverage the coupling effects of surface structures and materials to enable strong adhesion by regulating interfacial media behaviors and optimizing contact stress distribution, with broad applications in the precision medicine. [Pictures of Gecko were reprinted with permission from ref (6). Copyright 2006 The National Academy of Sciences of the USA. SEM of tree frog toe pads was reprinted with permission from ref (7). Copyright 2006 The Royal Society. Photographs of an octopus and its sucker were reprinted with permission from ref (9). Copyright 2017 Springer Nature. Diagram of negative pressure was reprinted with permission from ref (8). Copyright 2013 Authors, licensed under a Creative Commons Attribution (CC BY) license, published by PLoS One. The tree frog-inspired structure was reprinted with permission from ref (11). Copyright 2020 The Authors under a Creative Commons Attribution 4.0 International License, published by Wiley-VCH. The octopus-inspired architecture was reprinted with permission from ref (9). Copyright 2017 Springer Nature. The photograph of tissue adhesives was reprinted with permission from ref (30). Copyright 2019 Springer Nature. The photograph of drug delivery was reprinted with permission from ref (31). Copyright 2019 American Association for the Advancement of Science. The photograph of biosignal monitoring was reprinted with permission from ref (32). Copyright 2019 Wiley-VCH.]

## Evolution of Natural Biological Attachment Surfaces and the Underlying Mechanisms

**Figure 2:**
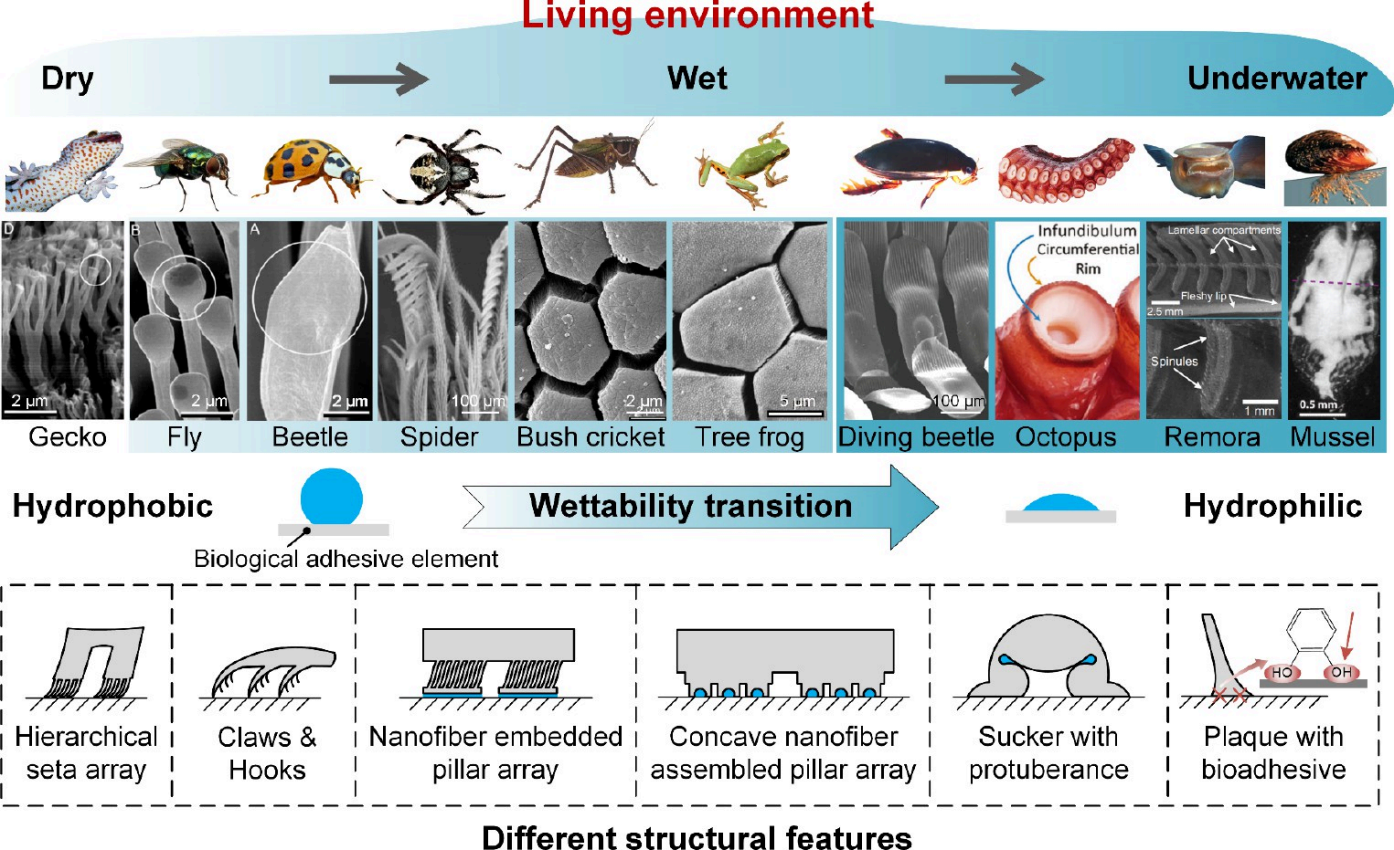
Overview of strong attachment organisms and their adhesive structures across terrestrial to aquatic environments. Organisms have evolved a variety of attachment structures to adapt to changing humidity conditions, from dry to wet environments. These range from the hierarchical seta arrays of geckos for dry adhesion, to the patterned pillar arrays of tree frogs for wet adhesion, to the suction structures of octopuses and the chemically adhesive structures of mussels. As environmental humidity increases, the wettability of biological adhesive elements transitions from hydrophobic to hydrophilic, reflecting a trend that correlates with the level of environmental humidity. [Pictures of the gecko, fly, and beetle were reprinted with permission from ref (18). Copyright 2003 The National Academy of Sciences. Pictures of the spider were reprinted with permission from ref (23). Copyright 1994 American Arachnological Society. Pictures of bush cricket were reprinted with permission from ref (21). Copyright 2004 The Society. Pictures of tree frog were reprinted with permission from ref (12). Copyright 2009 The Company of Biologists. Pictures of diving beetle were reprinted with permission from ref (22). Copyright 2014 Royal Society. Pictures of octopus were reprinted with permission from ref (32). Copyright 2018 The Authors under a Creative Commons Attribution 4.0 International License, published by Wiley-VCH. Pictures of remora were reprinted with permission from refs (24) and (25). Copyright 2012 Wiley-VCH. Copyright 2015 The Company of Biologists. Pictures of mussel were reprinted with permission from ref (26). Copyright 2011 Annual Reviews.].

Over billions of years of evolution, nature has produced numerous organisms with exceptional adhesive abilities. These organisms rely on unique tissues or organs to achieve stable adhesion and friction, offering valuable insights for human research on interface adhesion behaviors. Depending on the humidity of their living environments, biological adhesion can be categorized into dry adhesion, wet adhesion, and underwater adhesion (Figure 2). For example, geckos are adapted to dry conditions, crickets and tree frogs thrive in humid environments, while octopuses and mussels inhabit underwater environments. Organisms from different environments possess distinct adhesion structures, which can be classified based on their features into hierarchical seta arrays, claw and hook structures, micronano hierarchical pillar arrays, sucker structures, and adhesive plaques formed by protein deposition. Among these, the micronano hierarchical pillar array structure includes micropillar structures embedded with nanofibers and nanofiber self-assembled micropillar with pits. These structures achieve effective adhesion in complex environments through various mechanisms, such as van der Waals forces, capillary forces, mechanical interlockings, negative pressure suction, and chemical adhesion.

Furthermore, the wettability of the materials in biological adhesive units varies with the living environment, transitioning from hydrophobic to hydrophilic properties (Figure 2). It is therefore suggested that the interfacial liquid plays a crucial role in biological adhesion as conditions shift from dry to wet. Drawing inspiration from biological adhesion, researchers have conducted extensive studies on the underlying mechanisms and developed various bioinspired strong attachment surfaces. The following section will introduce the adhesion mechanisms of biological attachment systems across a range of environmental humidity and analyze the influence of micronano structures and materials on attachment, focusing on the regulation of interfacial media behavior and optimization of contact stress distribution.

### Mechanisms of Biological Attachment Surfaces

#### van der Waals Force for Dry Contact

van der Waals forces are long-range interaction forces between molecules, consisting of three distinct types: the induction force, the orientation force, and the dispersion force. Dispersion forces are the dominant component of the total van der Waals forces between atoms and molecules. These long-range forces remain effective over a wide range of distances, from greater than 10 nm to interatomic spacings of about 0.2 nm.^35^ Because dispersion forces are always present—unlike other types of forces, which depend on the specific nature of the molecules—they play a crucial role in many important phenomena, including adhesion, surface tension, wetting, strength of solids, and the behavior of gases and liquids.

**Figure 3:**
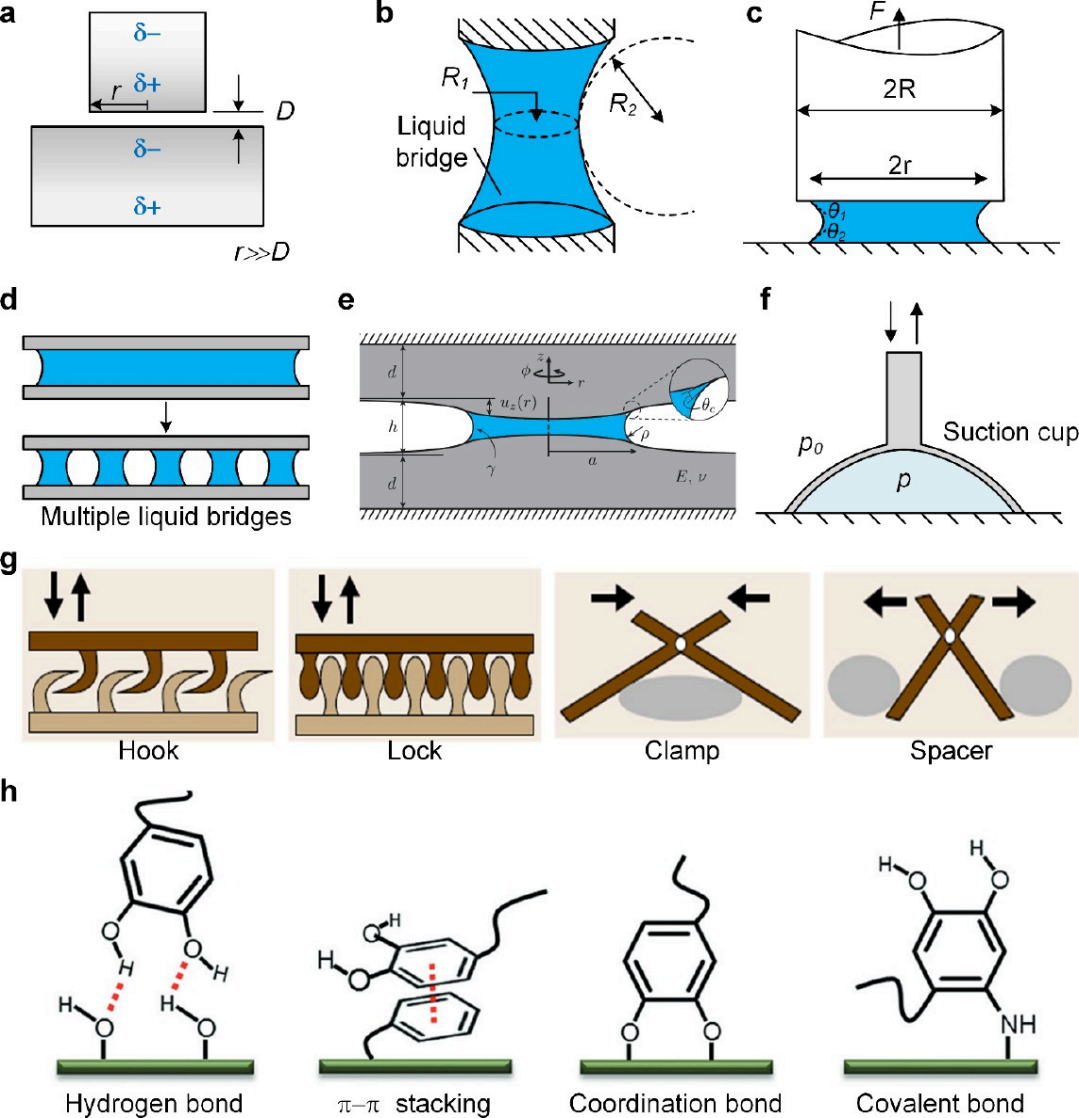
Physical and chemical mechanisms of biological attachment surfaces. (a) Schematic diagram of van der Waals force between two flat surfaces. (b) A liquid bridge with two principal radii of curvature between two solid cylinders. (c) Schematic diagram of a liquid bridge formed between a flat-ended fiber and a rigid substrate. Reprinted with permission from ref (51). Copyright 2006 Elsevier. (d) Schematic diagram of liquid bridge splitting. (e) Deformation of two elastic substrates induced by capillary force. Reprinted with permission from ref (54). Copyright 2014 American Physical Society. (f) Differential pressure between inside and outside the suction cup. (g) Four typical structures of mechanical interlocking. Reprinted with permission from ref (65). Copyright 2010 Springer-Verlag. (h) Multiple physical and chemical interactions of catechol groups in wet adhesion. Reprinted with permission from ref (86). Copyright 2018 Wiley-VCH.

In biological dry adhesion systems, such as gecko adhesion, van der Waals forces are a key component of the total adhesive force at the interface. The dispersion force between two flat surfaces per unit area is represented as follows:^36^
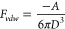
where *A* is the Hamaker constant, and *D* is the distance between two flat surfaces (Figure 3a). Hamaker constant *A*, which depends on the volume and polarizability of the molecules involved, typically falls within the range of 4 × 10^–20^ to 4 × 10^–19^ J for most solids and liquids, affecting the interaction strength by no more than a factor of 10. Therefore, the distance *D* plays a more significant role in determining the van der Waals interaction strength, as the strength is inversely proportional to the cube of the distance *D*.

**Figure 4:**
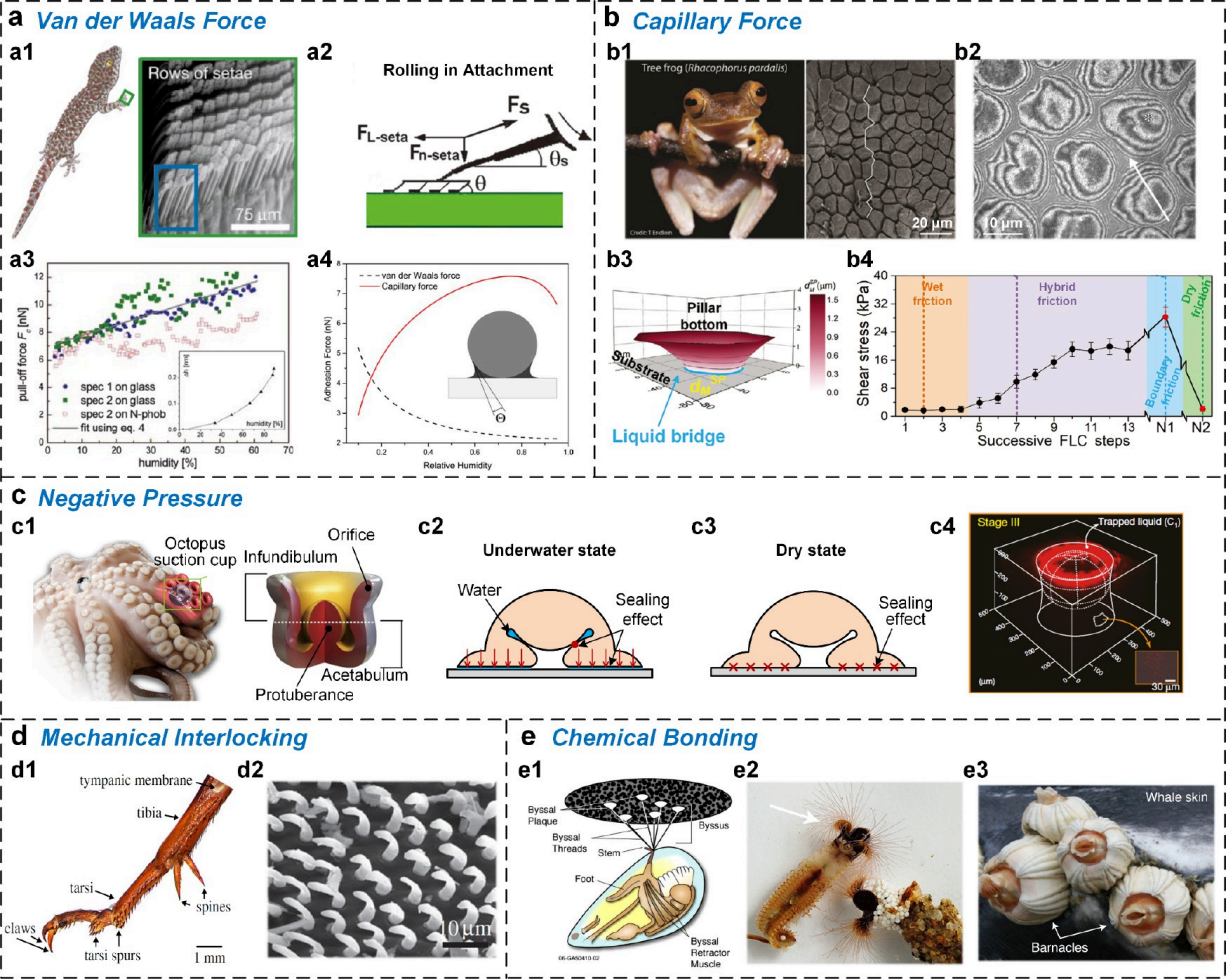
Attachment mechanisms of typical natural organisms. (a1) Photograph of a gecko and scanning electron micrograph (SEM) of its pad setae. Reprinted with permission from ref (37). Copyright 2000 Nature. (a2) Diagram of attachment of a single seta by rolling in the toes. Reprinted with permission from ref (6). Copyright 2006 The National Academy of Sciences of the USA. (a3) The adhesion force between the gecko spatula and substrates increases apparently with increasing humidity at ambient temperature. Reprinted with permission from ref (40). Copyright 2005 The National Academy of Sciences of the USA. (a4) Simulation of the variation in adhesion forces between a silicon nitride sphere and a mica at different relative humidities, including van der Waals and capillary forces. Reprinted with permission from ref (41). Copyright 2005 Elsevier. (b1) Photograph of a tree frog and SEM of its toe pads. Reprinted with permission from ref (55). Copyright 2015 Wiley-VCH. (b2) Characterization of the interfacial fluid layer between tree frog toe pad and glass substrate using interference reflection microscopy. Reprinted with permission from ref (7). Copyright 2006 The Royal Society. (b3) Deformation of a soft pillar surface caused by an interfacial liquid bridge. (b4) The friction of tree frog toe pads varies during successive frog-like-crawling (FLC) steps and reaches a maximum at the boundary friction. Reprinted with permission from ref (11). Copyright 2020 The Authors under a Creative Commons Attribution 4.0 International License, published by Wiley-VCH. (c1) Photograph of an octopus and schematic diagram of the sucker structure. (c2, c3) Illustrations of octopus suckers attached to substrates in underwater and dry states, showing the sealing effects induced by cohesive forces of water and van der Waals forces, respectively. (c4) Confocal fluorescence image showing the adhesion stage of the sucker, with liquid trapped in the upper chamber. Reprinted with permission from ref (9). Copyright 2017 Springer Nature. (d1) Photograph of a cricket leg. Reprinted with permission from ref (66). Copyright 2018 The Authors under a Creative Commons Attribution 4.0 International License, published by the Royal Society. (d2) SEM of ventral side setae in gill lamellae of mayfly larvae. Reprinted with permission from ref (68). Copyright 2010 Company of Biologists. (e1) Schematic drawing of a mussel attached to a substrate by byssal plaque. Reprinted with permission from ref (75). Copyright 2007 Springer. (e2) Photograph of sandcastle worms using adhesive proteins to glue sand grains together to build tubular shelters. Reprinted with permission from ref (76). Copyright 2018 Wiley-VCH. (e3) Barnacles adhered to the skin of a whale. Reprinted with permission from ref (78). Copyright 2021 Springer Nature.

In nature, the microscopic structure of a gecko’s foot is composed of millions of tiny, hair-like setae (Figure 4a, a1), which further divide at their tips into spatula-shaped nanostructures approximately 200 nm in width and 5 nm in thickness, creating a hierarchical micronano structure.^37^ These structures establish “close” contact with the substrate, generating an adhesion force several times greater than the gecko’s body weight. Autumn et al. characterized the adhesive force of individual seta and the entire foot pad, demonstrating that the primary adhesion force results from van der Waals interactions between the setae and the contacting surface.^38^ They found that setae adhesion is direction-dependent and explained the mechanism of gecko adhesion under dry conditions (Figure 4a, a2). Based on Autumn et al.’s measurements, a single seta can generate 194 μN in shear and 40 μN in adhesion when properly oriented, preloaded and dragged.^37^ All 6.5 million setae of a 50 g gecko could theoretically generate about 1,300 N of shear force, enough to support the weight of two humans.^39^ Therefore, the gecko’s adhesion system appears to be highly redundant. However, it is unlikely that all setae can achieve the maximum adhesion simultaneously, particularly when the gecko adheres to rough, contaminated, or wet surfaces. The setae structure ensures the stable gecko attachment in complex environments as well as in the presence of external disturbances during locomotion.

Although it is widely accepted that gecko adhesion is primarily influenced by van der Waals forces, some studies suggest the involvement of additional nonvan der Waals forces. For instance, adhesion to hydroxylated surfaces (such as glass and alumina) is notably stronger, while adhesion on hydrophobic-coated silicon wafers is weaker compared to bare silicon wafers. These results cannot be explained solely by van der Waals forces, which are insensitive to surface chemistry. Moreover, when water is present at the seta-substrate contact interface, it is believed to increase the distance between surfaces and reduce van der Waals forces. However, the diameter of a single water molecule (0.3 nm) remains well within the range of van der Waals attraction.^35^ Research by Arzt et al. showed that, at the scale of the setae, the adhesive force increases with humidity, and its relative contribution depends on both ambient humidity (Figure 4a, a3) and the hydrophilicity of the substrate, confirming that capillary forces also contribute to gecko adhesion.^40^ Sun et al. employed computer simulations to analyze the van der Waals and capillary forces between silicon nitride spheres and mica sheets at different relative humidities, finding that capillary forces dominate when relative humidity exceeds 16% (Figure 4a, a4).^41^ These studies suggest that van der Waals and capillary forces can synergistically enhance gecko adhesion and are not mutually exclusive mechanisms.^42,43^ Additionally, recent work by Singla et al. investigated the sapphire-setae contact interface using interface-sensitive spectroscopy and directly confirmed the presence of acid–base interactions at the contact interface, suggesting that gecko adhesion is not a residue-free system solely based on van der Waals forces.^44^

#### Capillary Force for Wet Contact

When a liquid meniscus forms between two lyophilic solid surfaces due to vapor condensation or adsorbed liquid, the attractive force acting perpendicular to the surfaces is the capillarity force.^45^ As is well-known, when the liquid surface is curved in equilibrium, a pressure difference, known as the Laplace pressure, exists between the inside and outside of the liquid. The Laplace pressure can be expressed as^46^
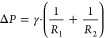
where *R*_1_ and *R*_2_ are two principal radii of curvature. In the absence of external fields (e.g., gravity), the Laplace pressure Δ*P* is the same throughout the liquid, and the curvature is constant across the liquid surface. By comparing the Laplace pressure to the hydrostatic pressure, a characteristic length scale, known as the capillary length, can be estimated as , which is generally on the order of few millimeters.^47^ For liquid bridges with radii of curvature below this characteristic scale, the effect of gravity can be neglected.

In biological wet attachment systems, such as those in insects, tree frogs, and arachnids, the structural dimensions of adhesive terminals typically range from hundreds of micrometers to nanometers, with interfacial contacts mediated by thin films of liquid secretion. The scale of the interfacial liquid film is no more than a few hundred nanometers,^7,48−50^ which is much smaller than the capillarity length κ, indicating the gravity can be ignored in the biological capillarity adhesion. In the case of a liquid bridge between a flat-ended fiber and a planar substrate (Figure 3b), as investigated by Qian et al., the total adhesive force *F* consists of two components: one induced by the Laplace pressure and the other arising from the axial component of liquid surface tension acting along the liquid perimeter at the tip of the fiber.^51^ The force *F* is expressed as follows:
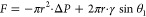
where *r* is the radius of the wet area on the fiber tip, γ is the liquid surface tension, and θ_1_ is the contact angle of the liquid with the fiber surface. The biological adhesive structural size, material surface wettability, and liquid surface tension all influence capillary forces. Theoretical analyses indicated that reducing the size of adhesive structures and improving the wettability of contact surfaces could enhance the wet adhesive strength until the liquid cavitation threshold is reached.^51^ Besides, the splitting of liquid bridge into multiple smaller bridges leads to an increase in capillary force.^52^ Furthermore, given the soft properties of biological adhesive pads, capillarity forces can deform adhesive pads to conform to contact substrates. The phenomenon is known as Elastocapillary effect.^53^ As predicted by theory and validated experimentally, this deformation, in turn, enhances the capillary force by increasing the contact area and decreasing the liquid film thickness, resulting in stronger adhesion.^11,54^

When liquid is present at the contact interface, friction is highly influenced by the state of interfacial liquid film states and exhibits a significant variation as the liquid volume decreases. In humid environments, amphibians such as tree frogs still exhibit strong crawling abilities on slippery surfaces, which are primarily attributed to the unique structural morphology of their toe pads and the interfacial liquid. The surface of a tree frog’s toe pad is covered with densely packed, microscale polygonal pillars, each approximately 10 μm in diameter, with groove depths and widths of about 5 and 1 μm, respectively (Figure 4b, b1).^55^ Each microscale pillar is densely packed with nanofibers about 250 nm in diameter, with pitted tips, forming a hierarchical micronano structure.^11^ Several studies have shown that the densely packed pillar structure effectively drains the excess interfacial liquid into the grooves, expelling it from the contact area and facilitating solid–solid contact, thereby increasing friction.^55,56^ Moreover, the toe pad continuously secretes mucus, which evenly covers its surface. Federle et al. used interference reflection microscopy (IRM) to measure the thickness of the fluid layer between a living tree frog toe pad and a glass substrate (Figure 4b, b2).^7^ They found that the distance between the center of the pillar and the substrate was the smallest, with the thinnest liquid film, ranging from approximately 0 to 35 nm. This nanometer-thick liquid film generates strong capillary forces, causing the pillar structure to adhere tightly to the substrate (Figure 4b, b3). This increases the contact area and results in high friction—referred to as boundary friction (Figure 4b, b4)—which significantly enhances the adhesion of tree frogs. Furthermore, the strong capillary force of this extremely thin liquid film may induce mechanical interlocking between the pad and the substrate.^57,58^ It has been proposed that when the liquid bridge is reduced to the molecular-level, its solid-like pressure-bearing capacity and excellent fluidity enhance vertical adhesion and facilitate the horizontal transfer of pressure.^59^ This suggests that the capillary force of the thin liquid film may indirectly enhance adhesion through other attachment mechanisms.^60^

#### Negative Pressure for Multienvironment Contact

Many organisms in nature, including octopuses, remoras, clingfish, golden algae eaters, suckermouth catfish, and loaches, utilize suction cups for attachment across diverse environments. While the sucker structures vary among species, their attachment mechanisms are based on creating a pressure difference between the inside and outside of suckers. Consequently, the adhesive force can be expressed as
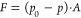
where *p*_0_ and *p* represent the external and internal pressures of the sucker, respectively, and *A* is the contact area of the sucker (Figure 3c). In terrestrial environments, *p*_0_ is the atmospheric pressure, and the pressure *p* inside the sucker can be regulated by contracting the muscles of the sucker chamber, resulting in a maximum adhesive strength of 101 kPa. While in underwater environments, *p*_0_ is the sum of atmospheric pressure and the hydrostatic pressure, and the pressure *p* inside the sucker can become negative due to the cohesiveness of water, which distinguishes it from terrestrial environments. As a result, the adhesive strength in underwater environments can exceed that achievable in terrestrial conditions. However, the internal pressure *p* cannot be reduced indefinitely. Once the pressure inside the sucker drops below a certain threshold, cavitation occurs within the water, leading to adhesion failure of the sucker. The cavitation threshold is primarily influenced by water purity, surface wettability, and ambient pressure.^61^ At sea level, cavitation normally limits the suction strength to a maximum of 200 kPa.^62^ Below sea level, where ambient pressure increases by 100 kPa for every 10 m of depth, the sucker can generate a greater pressure differential before reaching the corresponding increased cavitation threshold. Experiments on surfaces where cavitation would not be limiting showed a pressure differential ranging from 100 to 270 kPa, which is below the theoretical maximum.^62^ The discrepancy is due to the limited ability of suction cups to generate negative pressure.

Tramacere et al. observed morphological differences between various octopus species, particularly in the structure of the acetabular chamber.^13^ In some species, the acetabular chamber is a hollow, spherical cavity (such as the octopus suckers studied by Kier),^8^ while in others it is an ellipsoid with a rough protuberance (Figure 4c, c1). Based on this rough protuberance, researchers proposed a new underwater adhesion mechanism, suggesting that sustained adhesion is achieved by sealing the orifice opening between the protuberance and the infundibulum (Figure 4c, c2).^9^ The acetabular tissues of the sucker passively store elastic energy due to the cohesiveness of water in the infundibular cavity, which resists the expansive force of the acetabulum. Additionally, the loosely arranged epithelial cells around the infundibulum edge, along with the mucus they secrete, create an effective sealing effect.^8^ As a result, muscle contraction is no longer required to maintain low pressure in the cavity, allowing the sucker to adhere for extended periods. The researchers also employed histology, nuclear magnetic resonance, and ultrasound imaging techniques to reconstruct the 3D structure of the sucker and validate the adhesion model. Inspired by this mechanism, Baik et al. designed an artificial bioinspired sucker with a dome-like protuberance and used confocal fluorescence imaging to visualize liquid movement within the sucker’s chamber. During attachment and detachment, liquid flows through this orifice opening via capillary action (Figure 4c, c4). In dry conditions, the attachment is primarily governed by van der Waals forces due to the intimate contact between the infundibulum edge and substrate (Figure 4c, c3).^63,64^ Overall, octopuses achieve adhesion by adjusting the muscles of the sucker, reducing the pressure in the acetabulum and creating a pressure differential between the ambient pressure and the internal pressure of the sucker.

#### Mechanical Interlocking for Attachment

Mechanical interlocking relies on the adhesion and friction forces generated by the interlocking of biological attachment structures with the asperities or features on substrate surfaces, making it an effective attachment mechanism. Figure 3d illustrates a schematic diagram of the four common types of mechanical interlocking configurations identified by Gorb et al.^65^

This mechanism enables various organisms, including insects, arthropods, and fish, to achieve efficient adhesion. For instance, crickets and hornets use clawed legs and numerous small, sharp spines to crawl on rough surfaces (Figure 4d, d1).^66,67^ Dodds et al. provided a detailed comparison of how mayfly larvae adapt to both still and turbulent waters.^68^ They found that mayfly larvae achieve strong underwater adhesion through specialized attachment sites, such as the claws on their laterally directed legs, the setose pads on their gill lamellae (Figure 4d, d2), and the spikes on their abdomen.^69−71^ In teleost fish (*Garra getyla*), the tubercles with spines on the jaw sheaths may facilitate attachment through a mechanical interlocking mechanism.^72^ Additionally, Johal and Rawal proposed that the long hooks on the adhesive apparatus of hillstream fish (*Glyptothorax garhwali Tilak*) may also interact with the irregular matrix via this mechanism.^73^

It is noteworthy that attachment failures based on the mechanical interlocking mechanism are mainly caused by rupture, bending or yielding of either the attachment device or the substrate.^74^ Compared to van der Waals and capillary forces, mechanical interlocking is a relatively macroscopic form of adhesion.

#### Chemical Bonds for Attachment

Many organisms achieve reliable adhesion by secreting adhesives that form chemical bonds at contact interfaces. For example, mussels rely on adhesive proteins secreted by the byssus to form strong attachments to rough rocks (Figure 4e, e1),^75^ and sandcastle worms assemble protective tubular shells by secreting adhesives that glues sand grains or stones together (Figure 4e, e2).^76^ Barnacles secrete an adhesive known as “barnacle cement” that firmly attach their calcareous base plates to diverse underwater substrates, which is considered to be the most durable and hardest adhesion among aquatic organisms (Figure 4e, e3).^77,78^

Previous studies have shown that the primary functional component of the adhesives secreted by mussels and sandcastle worms is 3,4-dihydroxyphenylalanine (DOPA), which acts as a cross-linking agent and promotes interfacial adhesion.^76,79,80^ As shown in Figure 3e, DOPA mediates several interfacial interactions, including hydrogen bonding, π–π stacking, coordination bonding, and covalent bonding.^81−86^ At the bonding site, due to the competitive hydrogen bonding and dispersive force of the phenylene ring, the DOPA-based adhesive displaces preadsorbed water molecules from the substrate, thus facilitating bonding in aqueous environments, and finally curing for successful underwater bonding.^87−89^ Meanwhile, secreted adhesives can make nanoscale contact with substrates, forming mechanical interlockings that synergistically enhance attachment properties, similar to the situation in the attachment of creeper suckers.^90,91^

In conclusion, the above studies on the adhesion mechanisms of biological attachment systems have demonstrated that the attachment of organisms in different environments is closely related to their unique adhesion structures, materials, and interfacial media. It should be emphasized that the dynamic behavior of interfacial media regulated by micronano structures plays a crucial role in biological adhesion. In addition to adhesive materials, the development of artificial attachment surfaces should focus on the precise design and fabrication of micronano structured surfaces.

### Coupling Effects of Surface Micronano Structures and Materials on Interfacial Media and Stress Regulation

Building on studies of biological attachment mechanisms, significant progress has been made in the development of bioinspired strong attachment surfaces. Current research primarily focuses on designing micronano structures to improve interfacial attachment properties through two key aspects, as summarized in Table 1. The first is regulating the dynamic behavior of the interfacial liquid/air by precisely designing surface structures to enhance attachment via interfacial effects arising from the coupled interaction between structures and interfacial liquid/air media. The second is optimizing interfacial stress distribution by designing surface structures, such as adopting the “contact splitting” strategy and mushroom structures to improve attachment performance. The following section provides an overview of the current state of research in these areas.

**Table 1:**
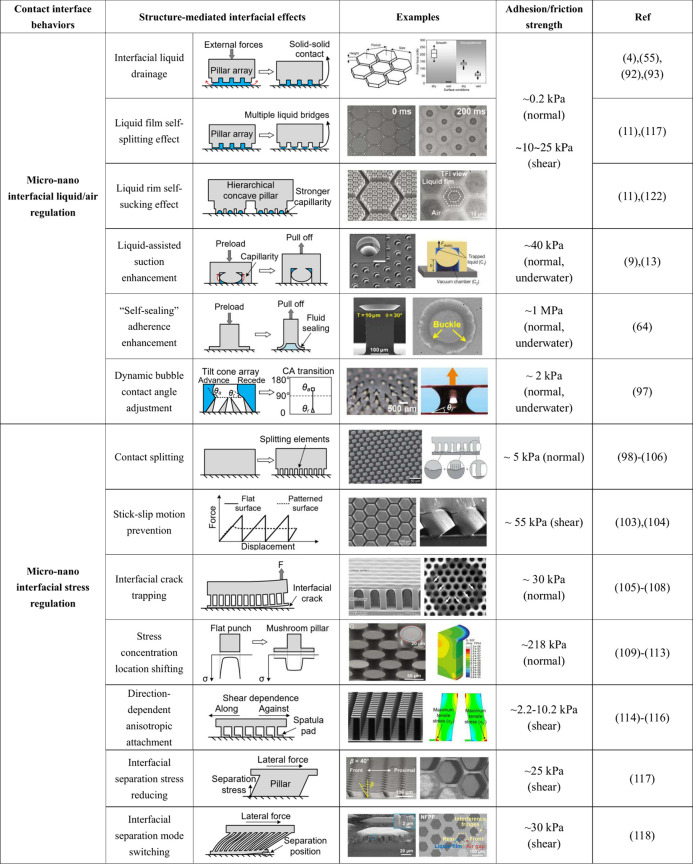
Summary of the Principles Governing the Influence of Surface Micro-Nano Structures and Materials on the Behaviors of Contact Interfaces

a Examples of interfacial liquid drainage were reprinted with permission from ref (93). Copyright 2009 Wiley-VCH. Examples of liquid film self-splitting effect and separation stress reducing were reprinted with permission from ref (117). Copyright 2022 Wiley-VCH. Examples of liquid rim self-sucking effect were reprinted with permission from ref (11). Copyright 2020 The Authors under a Creative Commons Attribution 4.0 International License, published by Wiley-VCH. Examples of liquid-assisted suction enhancement were reprinted with permission from ref (9). Copyright 2017 Springer Nature. Examples of self-sealing adherence enhancement were reprinted with permission from ref (64). Copyright 2022 The Authors under a Creative Commons Attribution License 4.0 (CC BY), published by American Association for the Advancement of Science. Examples of bubble adhesion were reprinted with permission from ref (97). Copyright 2021 Wiley-VCH. Examples of contact splitting were reprinted with permission from ref (102). Copyright 2016 Wiley-VCH. Examples of stick–slip motion prevention were reprinted with permission from ref (103). Copyright 2011 Royal Society of Chemistry. Examples of interfacial crack trapping were reprinted with permission from ref (108). Copyright 2007 The National Academy of Sciences of the USA. Examples of stress concentration location shifting were reprinted with permission from ref (111). Copyright 2021 Wiley-VCH. Examples of anisotropic attachment were reprinted with permission from ref (115). Copyright 2014 American Chemical Society. Examples of interfacial separation mode switching were reprinted with permission from ref (118). Copyright 2023 The Authors under a Creative Commons Attribution License 4.0 (CC BY), published by American Association for the Advancement of Science.

#### Bioinspired Interfacial Liquid/Air Regulation

Inspired by the structure of tree frog toe pads, various hexagonal arrays of prismatic structures were designed and fabricated.^55,92,93^ Under conditions where the interface is wetted by liquid, the patterned pillar structure effectively expels the excess liquid from the contact area, forming a thin liquid film at the interface. The capillary force generated by the thin liquid film enhances the interfacial solid–solid contact, which results in significantly higher friction of the patterned pillar surfaces compared to the planar surface. To investigate the effect of different polygon pillar patterns and sliding direction on wet friction, Zhang et al. prepared hexagonal, rhombic, and triangular pillar array surfaces.^27^ They found that the channels in pillar arrays influenced liquid movement during the sliding process, resulting in higher friction on the hexagonal pillar array compared to the other patterns, with minimal variation in friction with different sliding direction. Although various pillar surfaces have been designed to enhance wet attachment, their mechanisms typically rely on creating an interfacial liquid drainage strategy on single-level micropillar surfaces via external force, with little consideration given to hierarchical micronano structures and their coupled interactions with interfacial liquids. To further investigate the dynamic behavior of interfacial liquid on hierarchical structures, Zhang et al. proposed two-level concave micropillar surfaces inspired by tree frogs. *In-situ* observations of liquid movement revealed that the liquid self-splitting and self-sucking effects on two-level concave micropillars led to the formation of thinner nanometer-thick liquid films, which generate stronger capillary forces and boundary friction, thereby producing strong wet friction without external pressure.^11^ This study demonstrates how tree frogs use their unique micronano hierarchical structures to regulate interface liquid films. In addition, by utilizing the liquid surface tension, Vogel et al. achieved the adhesion and release of the planar substrate by controlling the expulsion and absorption of liquids on the pore array surface, corresponding to the formation and breakage of interfacial liquid bridges.^94^ Similarly, densely packed porous nanopillar surfaces proposed by Xue et al. also exhibited significantly increased adhesion in moist environments due to the capillarity-assistant formation of solid–solid contact.^95,96^

As mentioned earlier, the octopus can regulate the interfacial liquid behavior through their special sucker structure to achieve reliable attachment, which inspired the design of bionic suction surfaces. Baik et al. presented an artificial bioinspired sucker with dome-like protuberances, achieving repeatable adhesion under both dry and wet conditions.^9^ The adhesion strength increased rapidly with preload, as the deformation of the sucker under varying preloads triggered capillary flow of internal liquid. Using confocal microscopy, the researchers revealed that the adhesion enhancement mechanism was driven by liquid capillary flow. Wang et al. employed mushroom-shaped microsucker structures, converting water into “glue” for interface adhesion, greatly enhancing adhesion in wet environments.^64^ The adhesion strength significantly exceeded atmospheric pressure and was approximately an order of magnitude greater than that observed under dry conditions. The underwater adhesion of deformable microsuckers depends on the complex interactions between geometry, material elasticity, pulling speed, and fluid dynamics. Similarly, bubbles can also function as interface adhesives. Inspired by diving beetles, Wang et al. used directional tilted cone arrays to regulate the dynamic contact angles of bubbles, enabling repeatable underwater adhesion.^97^ This approach is essentially a form of controlling the dynamic behavior of interface liquids.

These studies highlight the significance of interfacial liquid/air behavior in enhancing attachment at contact interfaces, suggesting that exploiting multiple interfacial effects from the interactions between micronano structures and interfacial media can improve the adhesion properties of bioinspired surfaces.

#### Bioinspired Interfacial Stress Regulation

Inspired by bioadhesion, Arzt et al. proposed the concept of “contact splitting” based on the inverse relationship between the body mass of natural organisms and the scale of their adhesion elements, which is reflected in the evolutionary design of biological attachment systems.^18^ By splitting up the interfacial contact into finer subcontacts, attachment properties can be significantly enhanced. Extensive theoretical and experimental studies have been carried out to explore and validate this principle. Since each adhesion element functions independently without being influenced by adjacent units, “contact splitting” structures exhibit superior adaptability to substrate roughness, enabling better conformal contact on rough surfaces compared to uniform flat adhesion. Applying the “contact splitting” strategy, considerable fibrillar adhesives have been developed. A hierarchical micronano pillar array structure, fabricated with polymers and carbon nanotubes, was capable of providing compliance to rough substrates and enhancing adhesion and friction performance.^98^ Ko et al. utilized hybrid nanowire forests as highly versatile and reusable connectors, exhibiting strong shear adhesion strengths in both dry and wet environments. This is attributed to their high aspect ratios, which generate large contact areas in the engaged mode.^99−101^ Barreau et al. fabricated polydimethylsiloxane (PDMS) fiber arrays and systematically studied the impact of fiber geometry (diameter and height) on adhesion performance on rough surfaces.^102^ Their study revealed, for the first time, that the adhesion and nonadhesion states of the structure depend on the matching relationship between fiber geometry and substrate roughness. To maximize adhesion performance, the fiber dimensions must be appropriately tailored according to the substrate roughness.

It has also been shown that the “contact splitting” principle can eliminate the “stick–slip motion” typically observed during friction on smooth surfaces, enabling stable and smooth sliding on bionic patterned surfaces.^103,104^ Moreover, unlike smooth and continuous adhesion contacts, where an interfacial crack can easily propagate across the entire contact area once initiated, “contact splitting” attachment systems effectively halt crack propagation during interface separation. The initial crack results in the detachment of only a single contact element, releasing the elastic energy stored in that unit, which no longer contributes to further crack extension. Consequently, the crack must be reinitiated at each individual contact element—a mechanism known as “crack trapping”, in which discrete detachment requires significantly more energy than continuous detachment.^105−107^ Glassmaker et al. proposed a fiber array structure with a thin film covering the top. Experimental results showed that its adhesion energy was nine times greater than that of the flat control.^108^ This enhancement was attributed to the flexible thin film, which maximized the interfacial contact area while maintaining the stability of the fiber structure. Additionally, the high compliance of the film transferred little load to the crack tip during peeling, thereby realizing “crack trapping” and effectively halting the interfacial crack propagation.

The terminal geometry of “contact splitting” adhesion elements also plays a crucial role in adhesion performance. For example, research indicates that mushroom-shaped pillars provide higher adhesion than conventional pillars. Carbone et al. investigated the debonding mechanisms of flat punch pillars and mushroom-shaped pillars.^109^ For flat punch pillars, interfacial cracks initiate at the pillar edges due to local stress concentration and rapidly propagate, resulting in complete detachment. In contrast, optimized mushroom-shaped pillars exhibit a more uniform stress distribution across the contact interface. During separation, cracks occur at the center of the contact interface and gradually propagate outward, while the outer regions remain in contact until full detachment. This process requires higher separation stress, thereby enhancing the attachment performance of mushroom-shaped pillars. Afterward, Heepe et al. used high-speed cameras to capture the interface separation process of a single mushroom-shaped pillar, detailing the entire dynamic behaviors from crack initiation and propagation to complete detachment, providing convincing evidence for the uniform stress distribution of mushroom-shaped structures.^110^

In addition to the homogeneous material modulus and the symmetric geometry, the material modulus gradient and structural anisotropy of adhesive surfaces notably influences the contact interface stress distribution as well. In the case of material modulus gradient, inspired by the anisotropic material properties of tree frog toe pads, Xue et al. constructed two types of gradient elastic modulus pillar structures.^111,112^ These designs shifted the region of maximum interface separation stress from the outer edge toward the center and harnessed the negative pressure suction effect generated during separation, resulting in improved adhesion performance of bioinspired surfaces. Tian et al. proposed a mushroom-shaped structure with a soft shell and a rigid core.^113^ The soft shell reduced the stress singularity at the contact interface and increased the contact area, while the rigid core improved the structural stiffness. During separation, this configuration shifted the maximum interfacial separation stress toward the center of the contact region, effectively suppressing the peeling behavior at the interface. Regarding structural anisotropy, the micropillars with gecko-inspired spatula pads whose adhesive properties were strongly orientation-dependent.^114−116^ The spatula pads caused variations in the delamination position and the location of maximum tensile stress under different shear directions. Zhang et al. developed nonuniform pillar array surfaces featuring inclination and gradient structures, which effectively reduced the interfacial separation stress on pillars while enhancing lateral stress transmission along the surfaces.^117^ These modifications improved the stability of interfacial liquid films during friction, leading to better boundary friction performance. Inspired by the attachment pads of bush crickets, Guo et al. designed a novel nanofibrous pillar array covered with thin films (NFPF), which demonstrated stronger frictional performance compared to bulk pillar array, attributed to the unique interfacial stress shifting effect.^118^ During sliding, the deformation mode of NFPF shifted from compressing to stretching, with the initial separation position changed from the rear to the front side of the pillar, in contrast to bulk pillars. Such interfacial stress shifting effect largely decreased the separation stress and enhanced the stability of nanoliquid bridges, highlighting the strengthened coupling between structural deformation and the interfacial liquid film behaviors during shear.

It can be seen that by designing the material and structural characteristics of adhesive surfaces, the interfacial stress distribution can be modified, enabling the regulation of normal adhesion and tangential friction performance in bioinspired surfaces.

## Bioinspired Strong Attachment Surface Fabrication

The biological prototypes and adhesion mechanisms of biomimetic adhesive materials have been described in the previous section, and researchers have been inspired by living organisms and guided by different adhesion mechanisms to design biomimetic adhesive materials for various environments. Therefore, in this section, various typical preparation methods such as mold-assisted replication, 3D printing, self-assembly, and field-induced fabrication methods will be elaborated.

### Mold-Assisted Replication

The mold-assisted method is the most commonly used method for the preparation of biomimetic adhesive materials, which has been favored by many researchers due to its simplicity of preparation. The molding method utilizes predesigned molds (e.g., nanoparticles, polymer molds, *etc.*) to guide the growth or assembly of the materials, resulting in the formation of a variety of biomimetic adhesive materials with specific microstructures, such as flat, spherical, flat with rounded edges, mushroom, spatula and concave tips, *etc*.^119^

**Figure 5:**
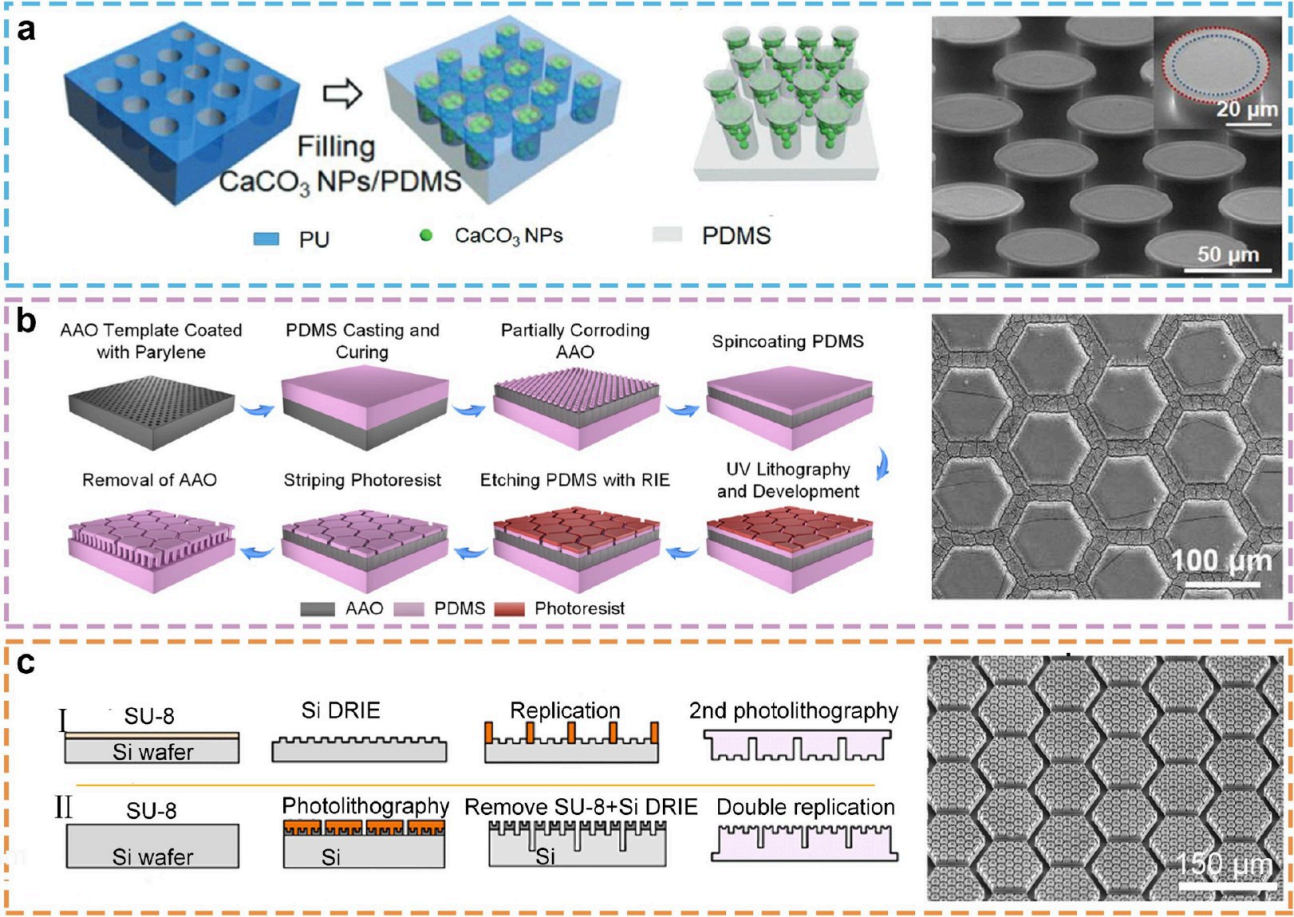
Adhesive structures prepared by mold-assisted replication. (a) The structure of mushroom tip prepared by a template method and its SEM images. Reprinted with permission from ref (111). Copyright 2021 Wiley-VCH. (b) Micronano structure of hexagonal prism prepared by a template method and its SEM diagram. Reprinted with permission from ref (118). Copyright 2023 American Association for the Advancement of Science. (c) Fractal hexagonal prism structure prepared by a template method and its SEM images. Reprinted with permission from ref (11). Copyright 2020 The Authors under a Creative Commons Attribution 4.0 International License, published by Wiley-VCH.

Inspired by geckos, researchers often use silicon as a mold to reverse the PDMS material to prepare adhesive materials with uniform columnar-shaped array structures to achieve surface adhesion.^120,121^ In addition, some researchers have added nanoparticles to PDMS to improve the overall adhesion properties of the material.^122^ The most representative approach is that of Xue et al., who employed a combination of a centrifugal method and a density difference between CaCO_3_ nanoparticles and matrix polydimethylsiloxane (PDMS) to achieve a gradient distribution of CaCO_3_ nanoparticles within a PDMS micron column, thereby enabling the realization of a gradient change in microcolumn modulus (Figure 5a).^111^ The work represents a significant advancement in the field of gecko-inspired dry adhesives, offering novel design principles and manufacturing techniques for the development of highly adhesive and friction-resistant materials. Moreover, the mold method is not solely employed for the fabrication of columnar structures. Campo’s team posited that the preparation of additional tip structures is feasible through the modification of mold parameters, including the angle, the presence of a contact surface, and so forth. These tip structures exhibit enhanced adhesion properties compared to their columnar counterparts. Moreover, the mold method can be employed to prepare micronano structures with diverse tips for dry adhesion, as well as to fabricate sucker structures for multimedia adhesion.^123^

The selection and design of the mold will affect the micro- and nanostructure of the material, which will in turn directly affect the adhesion property of the material. Moreover, anodized aluminum oxide (AAO) enables precise control over the dimensions and configuration of the aperture through alterations in voltage, current density, and other parameters. This structural adaptability allows for effective adaptation to diverse application requirements, making it a primary focus of research attention.^124^ In addition, the AAO mold exhibits high stability, a straightforward preparation process, and a relatively low cost, which collectively make it an optimal choice for large-scale production. Consequently, the use of AAO molds to prepare bionic adhesion micro and nanostructures has become the prevailing approach.^96,125−127^ The team led by Chen employed the AAO mold method to devise and construct a series of prismatic structure surfaces, to elucidate the influence mechanism of the bionic nanofiber prism array on wet friction (Figure 5b). Through in situ characterization of interfacial liquid film movement and prism deformation, it was determined that the bionic nanofiber prism has a distinctive interfacial stress transfer effect in wet friction, which enhances the stability of the prismatic interface nano liquid film. This results in the generation of stronger wet friction.^118^ While the AAO mold method offers high stability, it is important to acknowledge that the AAO mold itself is relatively brittle. Consequently, residual stress may be present in the material during the preparation of a micronano structure, which could potentially impact the material’s performance.

With the increasing demand for adhesion materials for the precision of micro and nanostructures, lithography has been applied to fabricate various bionic adhesive micro and nanostructures with high precision.^128,129^ In comparison to the AAO mold method, the lithography method demonstrates superior accuracy and repeatability and can be tailored to align with the requirements of applications that necessitate high precision. Moreover, lithography can be designed flexibly according to the specific requirements of the material in question and is not limited to a specific material system. Chen et al. employed lithography to fabricate an inhomogeneous prism array exhibiting tilt and gradient characteristics in height and width. Through in situ characterization of interfacial liquid film motion and soft prism deformation, it was observed that the liquid film underwent unique self-fragmentation, and contact stress was present on the surface of the inhomogeneous prism array for the first time. This provides theoretical support for the design of anisotropic strong wet friction bionic surfaces.^117^ Moreover, the team succeeded in preparing fractal hexagonal columnar micronanostructures through photolithography, reducing the two-stage microcolumn array on the toe pad of the tree frog, enhancing boundary friction, and elucidating the significance of liquid behavior induced by micronano structures (Figure 5c).^11^ Furthermore, lithography can facilitate the design of novel microstructures with enhanced adhesion properties.^130^ Hensel employed maskless two-photon lithography to devise a funnel-shaped micronano structure comprising a cylindrical configuration of airfoil. By modifying the angle of the airfoil, the adhesion of the micronano structure can be regulated, and the micronano structure displays enhanced adhesion in both dry and aqueous environments.^131^ While the photolithography method offers numerous benefits for the creation of biomimetic adhesion micronanostructures, it is important to acknowledge that, in comparison to other preparation techniques, the photolithography method often necessitates higher costs, more intricate processes, and greater energy consumption.

The mold-assisted method represents a conventional approach to the preparation of bioinspired adhesive materials. It enables the precise control of the dimensions, configuration, and structural characteristics of biomimetic adhesive micronano structure materials, facilitating an integrated synthesis. Nevertheless, the method also exhibits certain limitations, including a restricted range of suitable molds, the challenge of large-scale production, difficulties associated with molding, and the high cost of lithography. In practical applications, a comprehensive consideration of these factors is essential for the selection of the most suitable preparation method and process parameters.

### Two-Photon Polymerization Lithography

**Figure 6:**
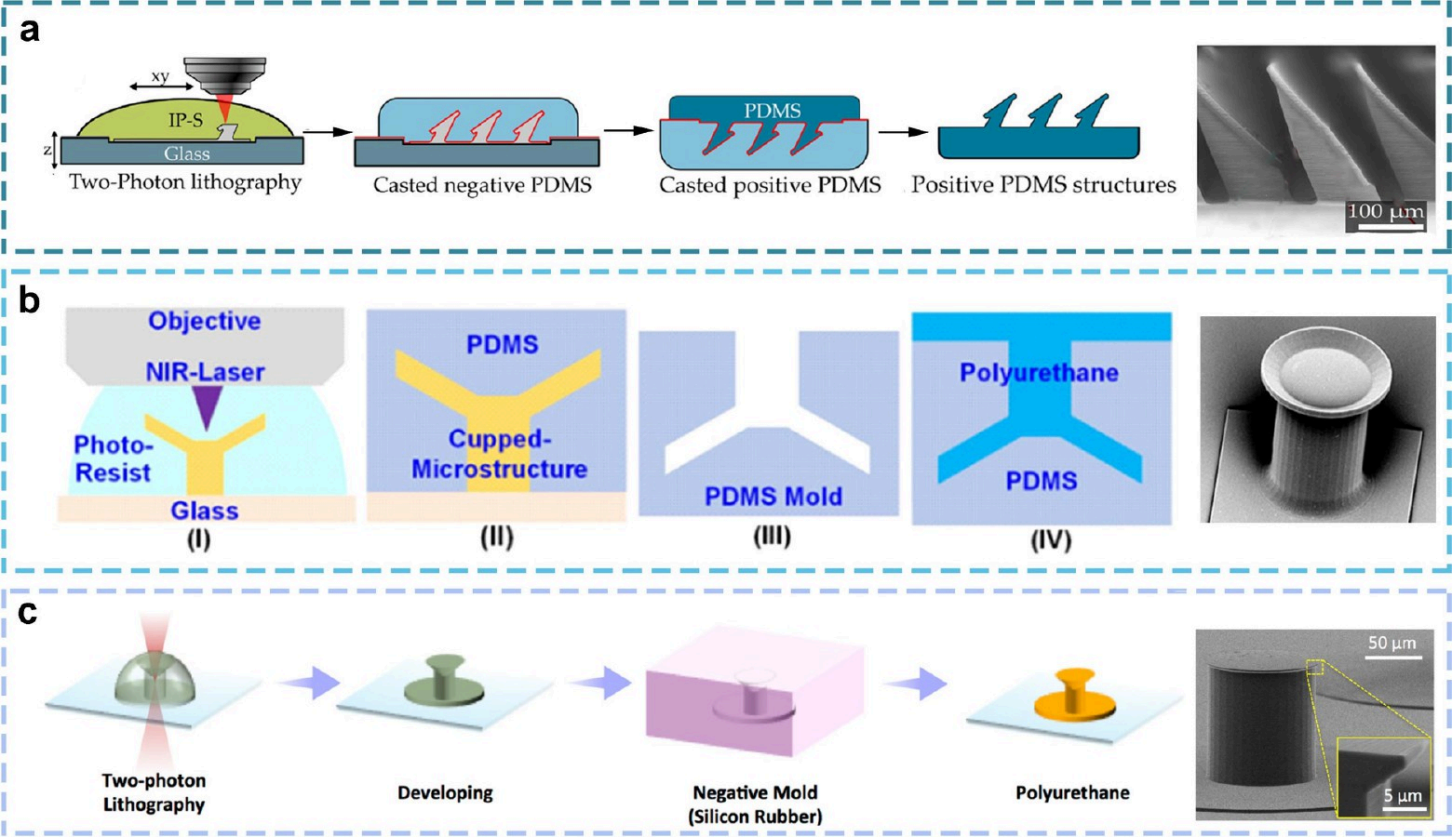
Adhesive structures prepared by two-photon polymerization lithography. (a) Schematic diagram of wedge structure prepared by two-photon polymerization photolithography and its SEM. Reprinted with permission from ref (133). Copyright 2020 Elsevier. (b) Schematic diagram of Winglike structure prepared by two-photon polymerization photolithography and its SEM. Reprinted with permission from ref (134). Copyright 2015 American Chemical Society. (c) Schematic diagram of mushroom structure prepared by two-photon polymerization photolithography and its SEM. Reprinted with permission from ref (135). Copyright 2021 Wiley-VCH.

Two-photon polymerization lithography (TPL) represents a nanoscale 3D printing technology that is capable of creating complex structures that transcend the limits of optical diffraction through a nonlinear two-photon absorption process in a liquid resin. The technology offers unparalleled processing capabilities, including the precise fabrication of micro- and nanoscale structures without alignment, as well as the rapid prototyping of highly complex three-dimensional nanostructures.^132^ The researchers prepared an anisotropic adhesive microstructure using polydimethylsiloxane (PDMS) as a material based on two-photon polymerization technology (Figure 6a). The resulting three-dimensional dry adhesive microstructure exhibited an anisotropic adhesion factor of 7.52:1, with a maximum adhesive strength of 1105.29 ± 40.93 mN/cm^2^ for glass.^133^

Maskless two-photon lithography offers a high degree of design flexibility, enabling the formation of micro- and nanoscale structures with a wide range of geometries. By employing this technique, the scientists devised a funnel-shaped micronano structure with an aerofoil column configuration (Figure 6b).^134^ By modifying the angle of the airfoil, the adhesion of the micronano structure can be regulated, and the micronano structure displays enhanced adhesion in both dry and aqueous environments. As a result, these funnel-shaped microstructures have great application potential on surfaces where wet and dry conditions require rapid conversion. However, the current mechanism of adsorption and detachment of cup-shaped microstructures is not perfect, and Hensel’s team based on two-photon lithography combined with the corresponding mathematical model created by the field pressure sensor and the observing system camera, describing the interaction between the attachment/detachment process, geometry, elastic fluid dynamics, and cup-shaped retractable velocity.^64^ Among the adhesion microstructures, the mushroom tip microstructure is the most attractive to researchers. This structure was prepared using polyurethane elastomers and the latest two-photon lithography technology by Sitti et al. The investigation of microstructure diameter and tip angle parameters has revealed that these variables influence the stress distribution and crack propagation of the microstructure. This represents the inaugural verification of the optimal design of the mushroom tip.^134^ Two-photon lithography represents a promising technique for the preparation of nanostructures suitable for dry adhesion. In addition, Hensel’s team has proposed a cup-shaped microstructure with a cavity inspired by the suction organs of aquatic animals (Figure 6c).^135^ The adhesion strength of this material on rough underwater surfaces can reach 200 kPa, which has significant potential for practical applications.^135^ In summary, two-photon polymerization photolithography offers the benefits of high resolution and the absence of a photomask. However, it is not without its constraints, including a high cost of equipment and a relatively slow processing speed.

### Self-Assembly

**Figure 7:**
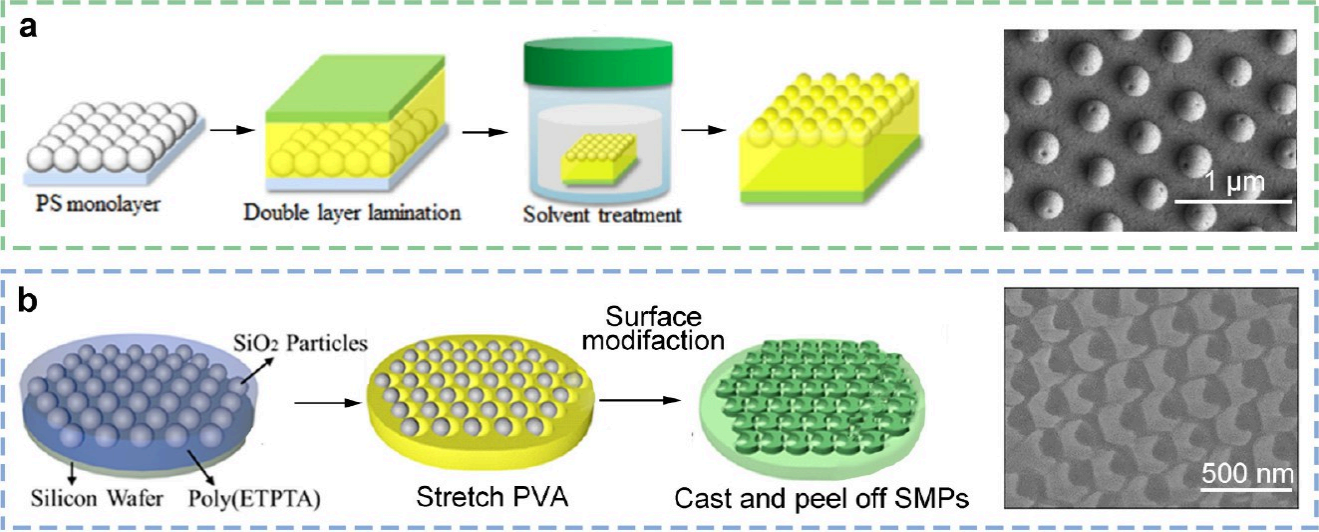
Adhesive structures prepared by self-assembly. (a) Self-assembly using PS microspheres. Reprinted with permission from ref (136). Copyright 2014 American Chemical Society. (b) Preparation of micronano suction cups by expandable colloid self-assembly. Reprinted with permission from ref (137). Copyright 2023 American Chemical Society.

The self-assembly method is also a more common approach for the preparation of bionic adhesive materials. This method often involves dispersing silica or polystyrene particles on the substrate and then casting with other materials. After demolding, an ideal micronano structure is obtained, resulting in a material with enhanced adhesion properties (Figure 7a).^136^ Yang et al. synthesized nanosuckers of crescent-shaped polyethoxylated trimethylolpropane triacrylate/polyethylene glycol diacrylate (ETPTA/EGDA) copolymers by integrating a scalable colloidal self-assembly technique and a similar soft lithography process (Figure 7b).^137^ The team also devised an array of structures that can generate arbitrary deformations and achieve long-term adhesion on irregular surfaces, including glass, sandpaper, and the pig’s kidney. Furthermore, nanosuckers that have developed adhesion deformation can be unglued by ethanol, and this reversible structure offers a potential solution strategy for areas such as wound care.

The self-assembly method possesses a unique flexibility that is not found in other preparation methods. The micromorphology and properties of the self-assembly structure can be modified by altering the type, size, and shape of the nanospheres. Moreover, the self-assembly technology does not necessitate the use of costly precision equipment or intricate process flows, thereby reducing the overall preparation costs. It is important to acknowledge that self-assembly technology also possesses inherent characteristics that warrant consideration. First and foremost, it is important to acknowledge the inherent limitations of self-assembly technology about the micromorphology of the target material. The interaction between nanospheres and the dynamic factors involved in the self-assembly process may result in a lack of complete control over the micromorphology and size distribution of the resulting structures. Second, the feasibility of large-scale industrial production remains a challenge for self-assembly technology. For instance, ensuring the uniformity and consistency of self-assembled structures in mass production is an urgent issue that requires resolution.

### Field-Induced Molding

**Figure 8:**
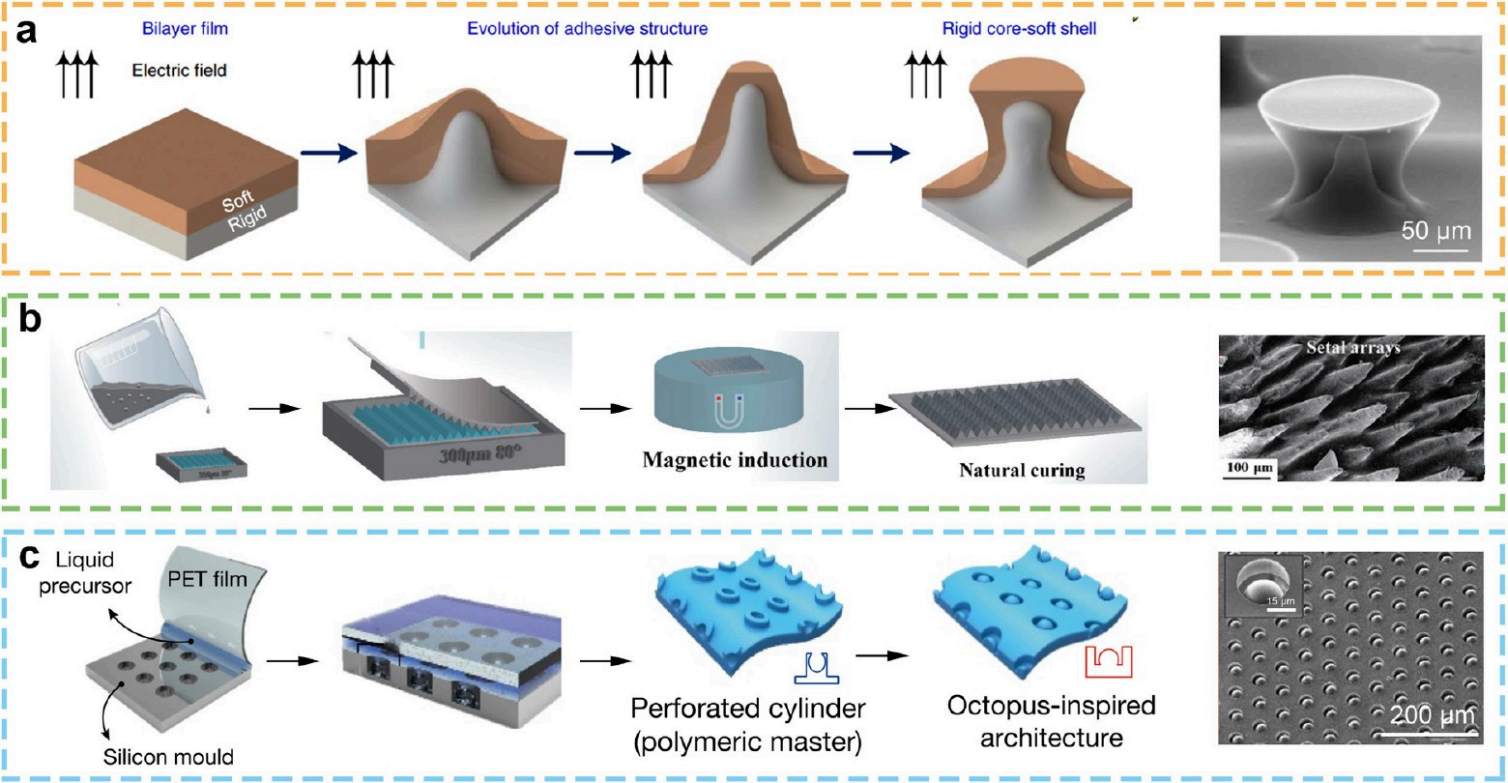
Adhesive structures prepared by field-induced molding. (a) The micro and nanostructures of the mushroom tip were prepared by electric field induction. Reprinted with permission from ref (113). Copyright 2022 Springer Nature. (b) The array of micro and nanostructures were prepared by magnetic field induced molding. Reprinted with permission from ref (139). Copyright 2023 Springer Nature. (c) Micronano structures of array suckers were prepared by flow field induced molding. Reprinted with permission from ref (9). Copyright 2017 Springer Nature.

The field-induced preparation of micro and nanostructures is a technique that employs electric or magnetic fields to influence the formation of specific micro and nanostructures in materials, including polymers and certain inorganic materials. The process of electric field induction typically entails the application of a voltage to the electrode pair, which generates an electric field. This, in turn, affects the molecular arrangement and flow behavior within the material, ultimately leading to the deformation and structuring of the material.^138^ Shao’s team has put forth a proposal for an electrically responsive self-growing core–shell structure. This structure, when acted upon by electrostatic field forces, forms a mushroom-like structure with a rigid core (bottom layer) and a soft curved shell (top layer) (Figure 8a).^113^ In comparison to alternative shapes, including flat, spherical, concave, and spade-shaped, the mushroom-shaped structure exhibits enhanced adhesion properties, overcoming the limitations of bionic dry adhesion structures in terms of target surface roughness and structural durability. And it offers a novel perspective for advancing research in the field of bionic dry adhesion. In addition to the electric field, which can induce the material to grow from the bottom up to micro and nanostructures, magnetic field induction is also a significant mechanism. Yang et al. fabricated a millimeter-level main layer structure and a micron-level sublayer cluster array based on a molding method and magnetic field-induced forming method (Figure 8b).^139^ The maximum adhesion strength was 5.1 kPa.

On this basis, the design of a magnetic adhesion surface can facilitate the stable adhesion and controlled release of various shapes of objects, which is anticipated to be applicable in automated industrial production lines, medical robots, and other fields. Pang et al. put forth a reversible wet/dry adhesion system via fluid induction, based on dome-like protrusions observed in octopus suckers (Figure 8c).^9^ The system fabricates a patterned structure from a polymer base material and utilizes it to create an opposing structure, notably without the necessity of designing a complex chemical synthesis or surface modification process. The micrometer-scale domes on the suckers demonstrate robust, reversible, and highly reproducible adhesion to silicon wafers, glass, and rough skin surfaces under diverse conditions, including dry, wet, underwater, and oil-covered surfaces. In short, the method of field-induced preparation of bionic adhesive materials offers the advantages of good controllability and high manufacturing efficiency. However, the equipment involved places higher demands, and not all materials can be prepared by this method. It is conceivable that in the future, the field-induced preparation of micro and nanostructures will extend beyond the preparation of a single structure, and will instead facilitate the integration and composite of multiple functions. For instance, the combination of additional technologies (such as chemical vapor deposition and lithography) enables the fabrication of micronano structured devices or systems with tailored functionalities.

**Figure 9:**
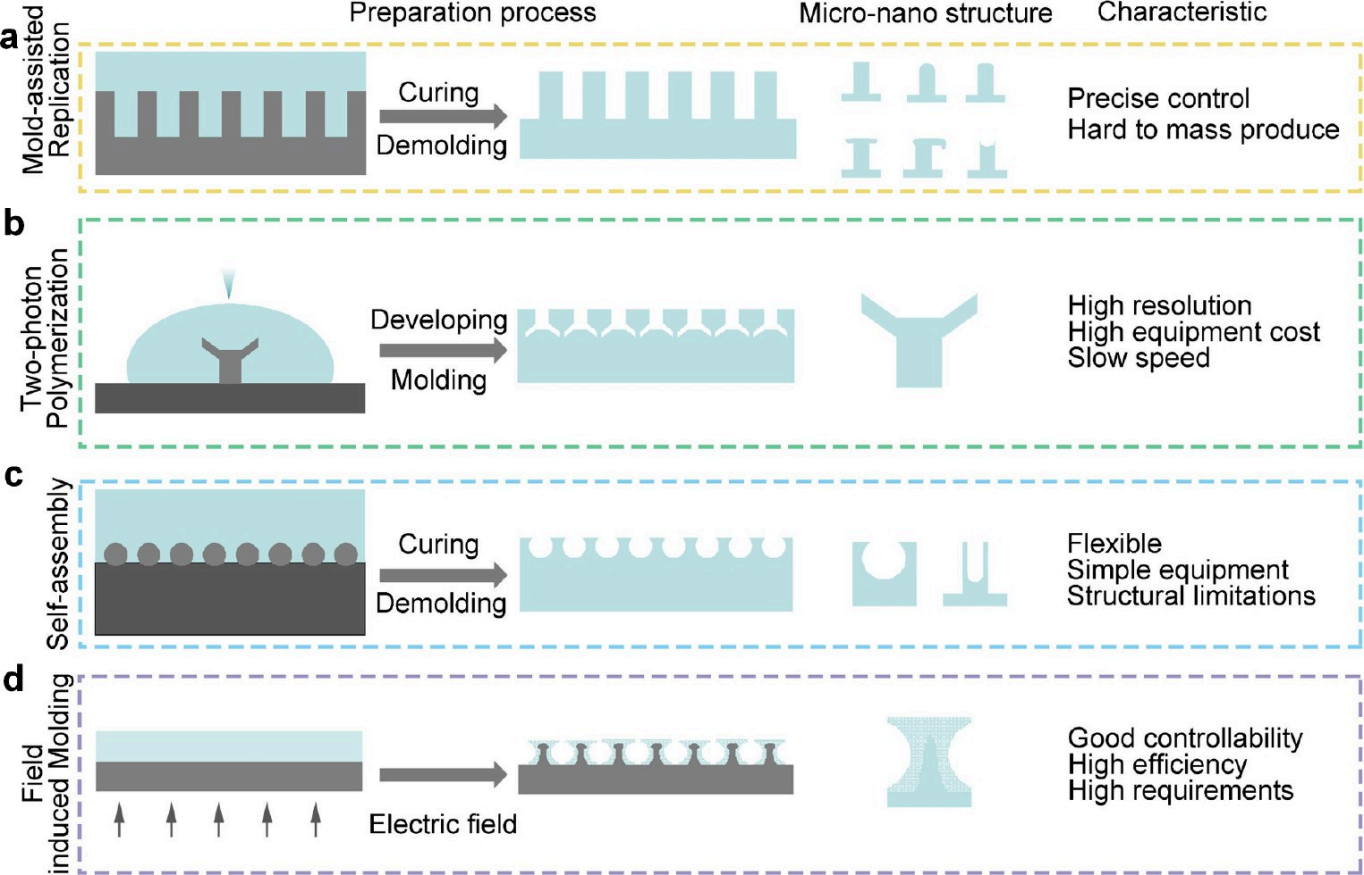
A Comparison of four different preparation methods. (a–d) refer to the preparation processes of the template method, the two-photon lithography polymerization method, the self-assembly method, and the field-induced molding method, respectively. This section also outlines the common microstructures prepared by these methods and their technical characteristics.

This section provides an overview of the most commonly used preparation methods. The mold method is the most prevalent approach utilized in the preparation of adhesive materials (Figure 9a). The use of an ordinary mold is inadequate for the preparation of materials with increasingly fine micro and nanostructures. Therefore, adhesion structures with different tips can be prepared by the AAO method. This method allows for the production of uniform nanocolumns; however, the residual stress on the AAO mold may impact the quality of the prepared material. To achieve greater precision, the lithography method is increasingly favored by researchers. However, the high cost of photoresist materials has the potential to significantly increase the financial burden associated with this preparation method, while also introducing additional complexity to the process. The Two-photon Polymerization Lithography allows for the preparation of micro and nanostructures with greater accuracy than other methods, although the degree of accuracy is not as high as that of the lithography method (Figure 9b). Additionally, the parameter changes that occur during the printing process may affect the properties of the material to a certain extent. The self-assembly method allows for the regulation of micronano structures through the alteration of nanospheres (Figure 9c). However, it may not be feasible to achieve complete control over the microstructure and size distribution of the resulting structure. The field induction method exhibits excellent controllability for the structure; however, it is not universally applicable and necessitates that the material responds to a specific physical field (Figure 9d). Therefore, an appropriate preparation method following the substrate to fabricate the requisite micro- and nanostructures and achieve optimal adhesion properties.

## Design and Application of Bioinspired Attachment Surfaces in the Medical Field

Biological tissues, as vital components of living organisms, manifest a wide array of physical and chemical properties. The modulus, in particular, serves as a pivotal indicator of the tissue’s rigidity and flexibility, ranging from soft visceral tissues to hard bones. The variation in modulus exerts a substantial influence on the selection and design of adhesives. Surface wettability, defined as the propensity of a surface to attract or repel water, is another critical factor influencing the adhesion process.

Chemical functional groups, representing another salient property of biological tissues, directly impact the interaction with adhesives by their type and number. These functional groups can influence the formation of chemical bonds, thereby enhancing the adhesive strength. Given these distinctive properties of biological tissues, the medical field imposes stringent requirements on adhesives. Specifically, adhesives must exhibit a modulus that is commensurate with that of the biological tissue to ensure stability and functionality at the bonding site. Additionally, the surface wettability of the adhesive plays a pivotal role in achieving a tight fit with the tissue surface, thereby enhancing the bonding efficacy. The adhesive must demonstrate compatibility with the chemical functional groups present in the biological tissue to ensure the formation of a robust chemical bond. These stringent requirements are meticulously designed to ensure the safety and efficacy of adhesives in medical applications.

### Surgical Applications

**Figure 10:**
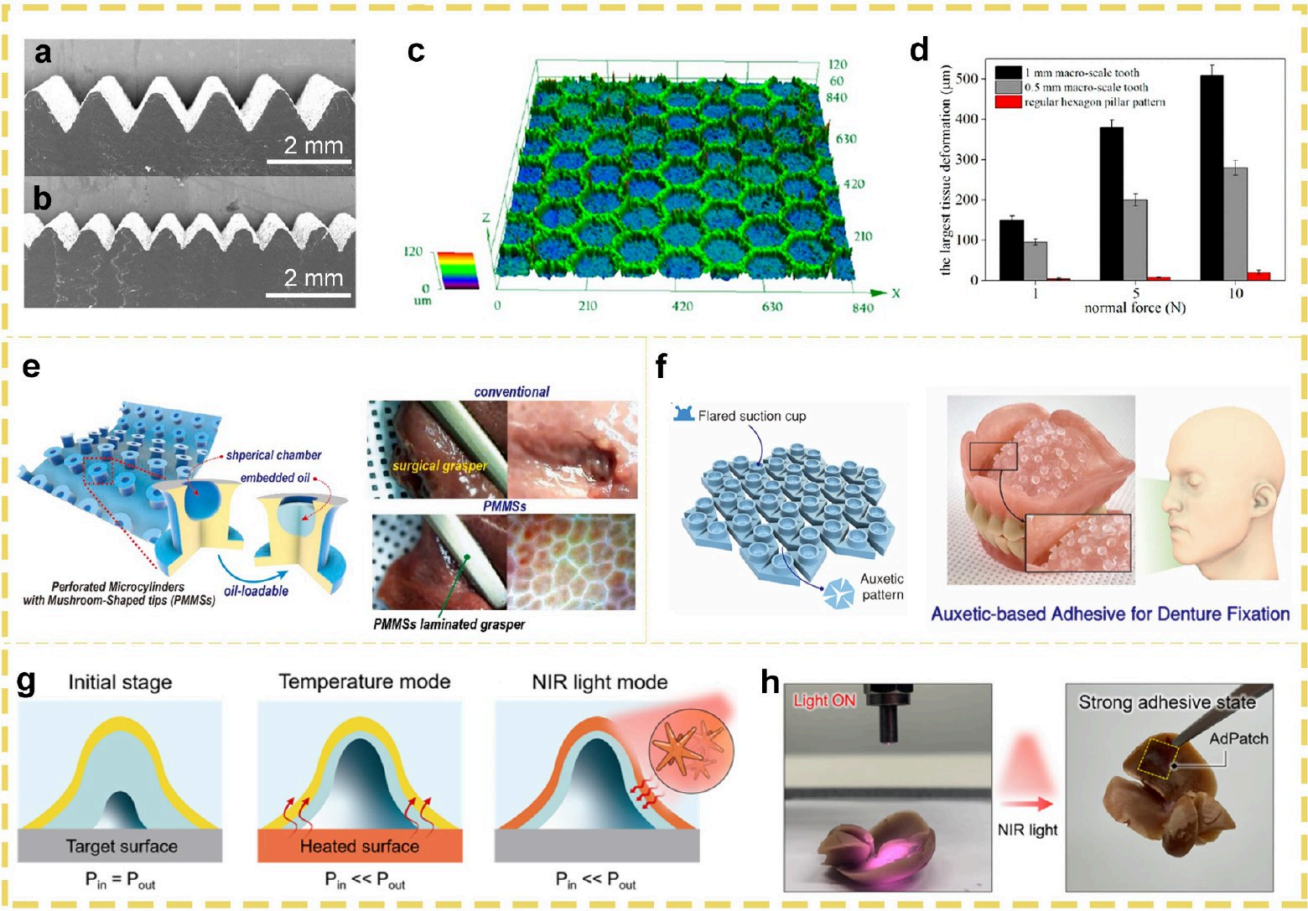
Bioinspired attachment structures for surgical grippers. (a, b) are photos of 1 mm and 0.5 mm dental grips, respectively. (c) The deformation of fresh pig liver when 10 N normal force is applied to the surgical instrument with a hexagonal column pattern. (d) Comparison of the largest tissue deformation induced by 1 mm and 0.5 mm macroscale teeth and hexagonal pillar pattern under normal forces of 1, 5, and 10 N. Reprinted with permission from ref (27). Copyright 2015 American Chemical Society. (e) Schematic illustration of perforated microcylinders with mushroom-shaped tips and snapshots of porcine liver grasping using a conventional surgical grasper and PMMS, respectively. Top-view snapshots show severe damage, while bottom-view snapshots demonstrate no significant damage when using the proposed PMMS. Reprinted with permission from ref (142). Copyright 2021 Elsevier. (f) A sucker structure with an origami process is used for artificial denture adhesion. Reprinted with permission from ref (144). Copyright 2024 American Chemical Society. (g) Adhesion mechanism of plasma adhesive patch under normal temperature, high-temperature environment, and infrared irradiation. (h) Photograph showing high adhesion on the mouse liver under NIR light irradiation (85 mW·cm^–2^, 3 min) (strong adhesive state). Reprinted with permission from ref (145). Copyright 2024 American Chemical Society.

Surgical grippers are used to grasp soft tissues and organs during surgery and are one of the most popular surgical instruments. However, there are risks associated with the use of grippers, as too little force can lead to soft tissue slippage and too much force can lead to soft tissue damage.^140,141^ To address this issue, researchers have proposed the integration of pressure sensors into surgical instruments. However, it is regrettable that soft tissue damage is an unavoidable consequence. It is therefore imperative that medical and surgical instruments can achieve a strong grip with minimal damage. Chen et al. designed a hexagonal columnar surgical grasper based on the structure of a tree frog toe pad (Figure 10c) and compared the deformation of soft tissues when this grasper was acted on with 1 mm and 0.5 mm (Figure 10a,b) tooth-like grippers (Figure 10d).^27^ The test results demonstrated that the gaps between the dental grippers were nearly filled with tissue when the normal force reached 10 N. In contrast, the hexagonal post grippers exhibited a depth of filling of approximately 30 μm. These findings demonstrated that the dental grippers resulted in significant soft tissue deformation and were ineffective in preventing damage, whereas the hexagonal post grippers were capable of achieving a secure grip while minimizing tissue damage. This phenomenon can be attributed to the hexagonal prism structure of the tree frog, which forms the boundary friction with the tissue surface. Concurrently, the stress acting on the soft surface is well dispersed, thereby reducing soft tissue damage.

Drawing inspiration from octopus, Pang et al. developed perforated microcylinders with mushroom-shaped tips (PMMS). The proposed bionic adhesive exhibited enhanced adhesion to both dry and wet skin, as well as to organ surfaces. This enhancement can be attributed to the capillary-assisted suction effect and the all-around shear resistance originating from structural and material properties. (Figure 10e).^142^ Moreover, the team employed an identical structure to fabricate an artificial octopus sucker (AOS) with soft microdenticles imprinted on the contact interface, which can adapt to surfaces that are highly rough or curved under the combined effect of negative pressure adsorption and capillary forces.^143^ Subsequently, the team proposed wet adhesive materials for application on oral prostheses, where the origami structure with negative Poisson’s ratio synergizes with negative pressure adsorption to alleviate stresses induced by tensile strains (Figure 10f).^144^ This reduction in stresses induced by curved surfaces makes possible the establishment of conformal contact with surfaces. In addition, the negative pressure structure of octopuses has provided a source of inspiration for the development of adhesive patches with elastic nanopore patterns. These patches exhibit adhesion that responds to temperature changes and near-infrared (NIR) light.^145,146^ When exposed to heat or light, the hydrogel undergoes a shrinkage, enabling the patch to achieve strong suction adhesion (134 kPa at 45 °C and 71 kPa at 85 mW·cm^–2^) (Figure 10g), and has been utilized for the transfer of ultrathin films and biosensors to fragile organs without causing damage (Figure 10h).^145^

The incorporation of adhesive materials with micronano structures into medical devices has been shown to yield several notable advantages. These materials exhibit enhanced wet adhesion properties, demonstrate exceptional deformation resistance, minimize damage to soft tissues, and offer novel design concepts for surgical instruments.

**Figure 11:**
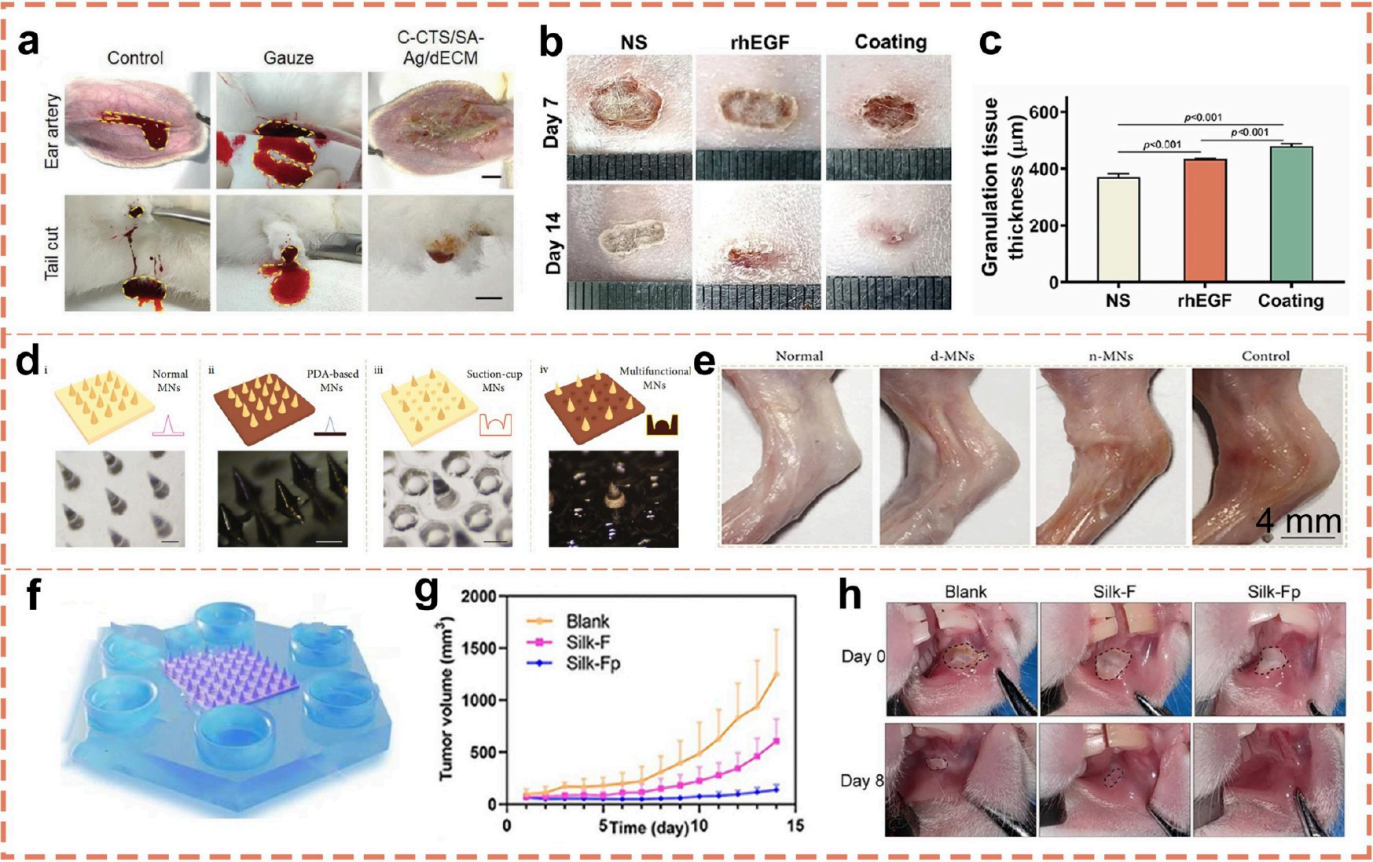
Bioinspired adhesive patches for wound closure and drug delivery. (a) Gross images showing hemostatic effects of the gauze and C-CTS/SA-Ag/dECM hydrogel with no treatment as a control (scale bars = 1 cm). Reprinted with permission from ref (150). Copyright 2022 Wiley-VCH. (b) Schematic illustration of the diabetic rabbit model, with quantification of wound contraction at days 7 and 14. (c) Granulation tissue thickness. Reprinted with permission from ref (153). Copyright 2024 American Chemical Society. (d) Four different structures: the normal group, the d-MN group, the n-MN group, and the control group, respectively. (e) Digital images of knee joints of rats in different treatment groups. Reprinted with permission from ref (160). Copyright 2020 The Authors under a Creative Commons Attribution 4.0 International License, published by American Association for the Advancement of Science. (f) Hydrogel microneedle suction cup drug delivery platform structure. (g) Average tumor growth curves of the tumors receiving treatments of Silk-Fp patch, Silk-F patch, and no treatment. (h) Gross inspection of buccal mucosa ulcers in rabbits treated with Silk-Fp patch, Silk-F patch, and no treatment at days 0 and 8. Reprinted with permission from ref (161). Copyright 2023 The Authors under a Creative Commons Attribution 4.0 International License, published by American Association for the Advancement of Science.

For traumatic or surgical injuries, hemorrhage control and wound closure are necessary to minimize life-threatening complications. Invasive methods of suturing and stapling are still commonly used; however, they are often time-consuming and can cause unwanted secondary medically induced injuries.^147−149^ It is therefore crucial for researchers to address the urgent need for tissue repair materials that exhibit strong adhesion to tissue and promote rapid wound healing. Yang’s team has introduced silver nanoparticles and decellularized ECM into hydrogels. The multinetwork hydrogels exhibited strong wet tissue adhesion (151.40 ± 1.50 kPa) due to several factors. The hydrogel demonstrated a rapid cessation of bleeding within 20 s, a result that was more pronounced than that observed in the untreated (control) and gauze groups (Figure 11a).^150^ Moreover, the gel possessed good biocompatibility and could be employed as an effective hemostatic material to achieve *in vivo* hemostasis.

In response to the growing demand for wound closure, researchers are dedicated to the development of innovative materials for tissue repair with robust adhesion, favorable compatibility, and the acceleration of wound healing. The aforementioned results indicate that the adhesive hydrogel has the potential for use in tissue sealing and repair. However, there is also a real-life hard-to-heal wound that continues to ooze and cause failure of the adhesive material, which places new demands on tissue repair materials for strong adhesion and functionality.^151,152^ Liu’s team prepared a Ca^2+^-induced rapid assembly of coating material into an extracellular matrix-like material based on scallop pedicle filaments.^153^ The team fabricated a diabetic rabbit and placed the coating material on its wound, and the results showed that the wound was able to achieve 90% contraction at 14 days (Figure 11b), and there was a great increase in granulation structure and collagen content (Figure 11c), which significantly promoted the wound repair, and achieved the treatment of difficult-to-heal wounds by modulating the microenvironment of the wound. In addition, researchers have been inspired by the structure of octopus negative pressure suction cups to create gel films and suction cup microstructures. These have been produced by template replication and mask lithography to enhance adhesion on wet surfaces while improving their biocompatibility and ability to promote wound healing.^154^ Hong et al. proposed that compositing the suction cups with a poly(acrylic) hydrogel composed of gelatin can provide strong adhesion in wet environments, with temperature stimulation to achieve switchability of adhesion. The efficacy and safety of the patch in surgical applications were validated through mechanical testing, adhesion assessment, and detailed biocompatibility analysis.^155^

Recovery of wounds often involves a lengthy recovery process, and as a result, many methods have been investigated to promote wound healing. Although many oral drug delivery vehicles have been developed currently, their complex design and low adherence time have limited their widespread application.^156−159^ Microneedles stand out in the field of drug retardation with the advantages of being noninvasive and painless, but most of them have a simple release strategy, which makes it difficult to achieve controlled active drug delivery. Based on this dilemma, Zhao et al. successfully prepared hierarchical microneedles with multifunctional adhesive and antibacterial abilities.^160^ It is noteworthy that each microneedle is encircled by a ring of concave chambers that contain dome-shaped protrusions. This configuration, reminiscent of the tentacles of an octopus, enhances adhesion capacity through a synergistic effect of negative pressure adsorption and mechanical interlocking. Consequently, the nanoneedles exhibit adhesive properties in dry, wet, and humid environments, achieving optimal drug slow-release performance. Moreover, tests showed that the microneedle array patches had high adhesion ability under different environments. Treatment experiments on mice with arthritis demonstrated that microneedles loaded with the corresponding drugs (d-MNs) exhibited a notable therapeutic effect on the joints of mice, as compared to the blank control (n-MNs) (Figure 11d,e).^160^ These results demonstrate that this bionic multifunctional microneedle has the potential to overcome the limitations of traditional microneedles and offer a promising avenue for broad applications in the field of universal, wearable transdermal drug delivery. Subsequent research by He et al. led to the development of a temperature-controlled multistage drug-release microneedle inspired by the blue-ringed octopus (Figure 11f).^161^ In this advanced microneedle technology, light-cured 3D printing technology is used for precision manufacturing, which ensures that the micronano-scale precision and mechanical strength of the microneedle structure are optimized. The design of the microneedle incorporates the principle of mechanical interlocking, enabling the active injection function of the microneedle to be activated during the preinjection phase after piercing the skin, relying on body temperature as the trigger mechanism. Once the conditions are met, the microneedle automatically switches to the drug release mode, which ensures that the drug is delivered to the target tissue in a smoother and more sustained manner through a carefully regulated release mechanism, thus significantly extending the duration of efficacy. In addition, the bionic suction cup structure enhances the stability and durability of the microneedle in complex physiological environments. The suction cup is based on the principle of negative pressure adsorption, and its adsorption ability in humid environments (with pressure greater than 10 kPa) has been optimized to ensure that the microneedle can be firmly adhered to the surface of the tissue, and achieve long-term stable retention, even in dynamically changing environments within the body. It not only effectively inhibits the growth and spread of tumor cells, but also greatly reduces the inconvenience and risk associated with frequent drug administration, while achieving anti-inflammatory effects in a short time and shortening the healing time of mouth ulcers (Figure 11g,h), providing strong technical support for precision medicine and chronic disease management.

For an extended period, hydrogels with high adhesion strength have been the primary materials employed for achieving tissue repair. Consequently, the mechanical properties of the gels warrant extensive attention. Second, numerous additional complications are frequently encountered during the process of tissue repair, including bacterial infections and inflammation. These pose significant challenges for the design of tissue adhesion materials, necessitating the preparation of multifunctional and integrated adhesion materials as a future development direction. The biocompatibility, cost, and multifunctional sustainability of adhesive materials must be taken into account during the preparation of such materials. Interdisciplinary research and collaboration must continue to facilitate the development of high-level tissue repair materials.

### Flexible Electronics

**Figure 12:**
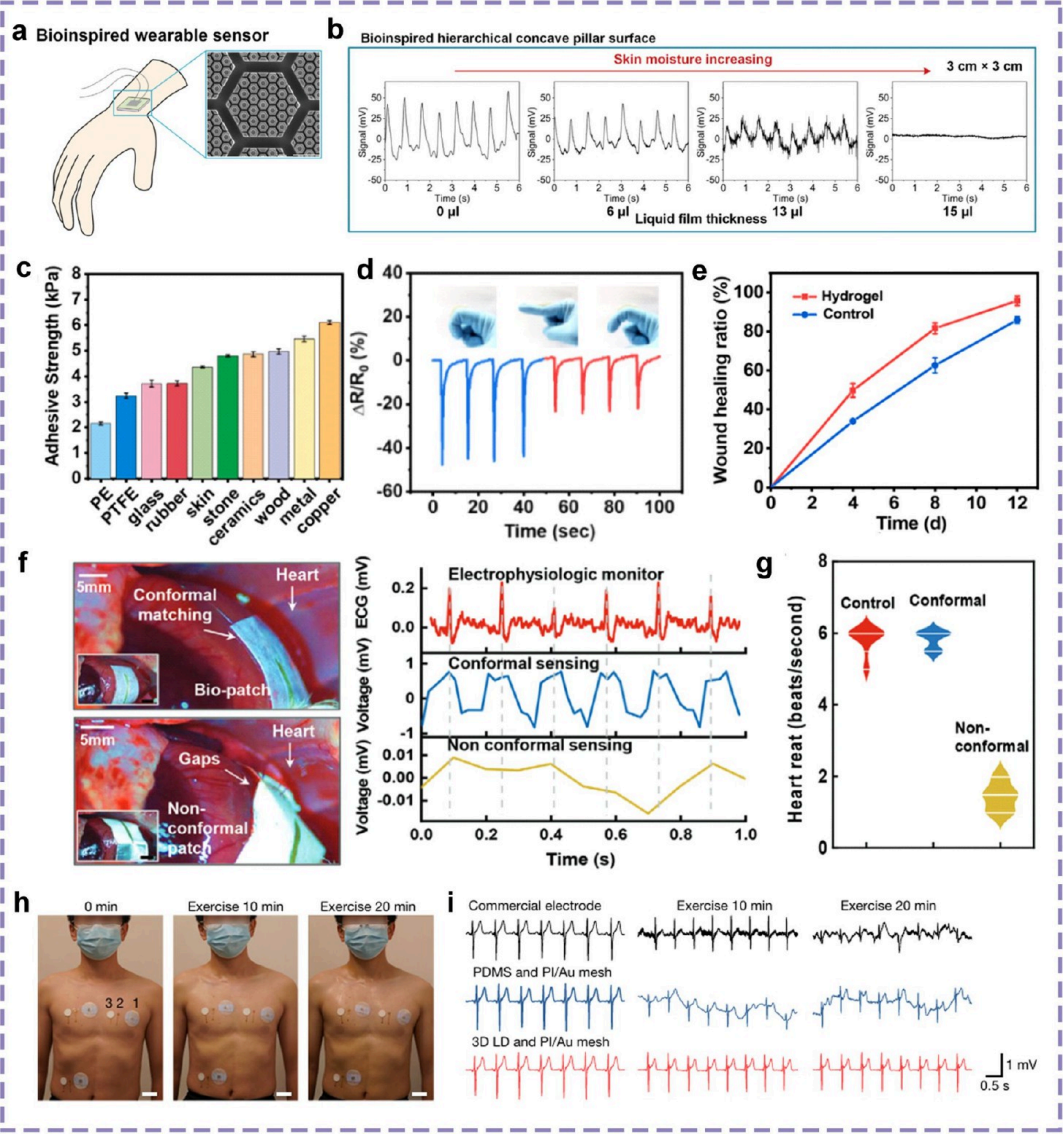
Bioinspired adhesive surfaces for flexible sensor adhesion interfaces. (a) Flexible electronic sensor with fractal hexagonal microcolumn structure. (b) Changes of pulse signal under different volumes of sweat. Reprinted with permission from ref (11). Copyright 2020 The Authors under a Creative Commons Attribution 4.0 International License, published by Wiley-VCH. (c) The adhesive strength of the bilayer composite hydrogel to different substrates. (d) Detect changes in the signal as the finger moves. (e) Statistics results of wound healing rate. Reprinted with permission from ref (164). Copyright 2021 Elsevier. (f, g) Comparison of the heart signals measured by an electrophysiologic monitor, the piezoelectric thin film, and the Biopatch, respectively. Reprinted with permission from ref (165). Copyright 2024 Wiley-VCH. (h) Images show commercial electrodes (1), PDMS/PI/Au mesh electrodes (2) and 3D LD/PI/Au mesh electrodes (3) on the skin before and after exercise. Scale bars, 5 cm. (i) ECG signals recorded from different electrodes before and after exercise. Reprinted with permission from ref (166). Copyright 2024 Springer Nature.

Early detection of fatal diseases is essential for medical diagnosis and treatment, and continuous and real-time monitoring has a positive effect on better management of patients suffering from chronic diseases, including cardiovascular diseases, diabetes and neurological disorders.^162,163^ Therefore, wearable devices show great potential in the medical field due to their deformability and compliance. Inspired by tree frogs, Chen et al. found that structures with fractal hexagonal micropillars have greater adhesion capacity, a feature that could be well suited for use in precision medicine.^11^ To this end, the researchers prepared a flexible electronic sensor with a fractal hexagonal micropillar structure (Figure 12a) to overcome the problem of ordinary sensor signals being affected by sweat, which flexible patch can detect pulse signals in the presence of 13 mL of sweat (Figure 12b). The hexagonal prism structure has been demonstrated to enhance the contact area between the footpad and the liquid surface. In wet environments, the hexagonal prism structure has been shown to spontaneously break up the liquid film to form extremely strong capillary adsorption, thereby enhancing the friction between the footpad and the slippery surface. Moreover, the nanopits present at the summit of the hexagonal column serve to augment the efficacy of capillary action, thereby yielding a patch that exhibits a fractal hexagonal column structure. This configuration affords enhanced adhesion and ensures that the test signal remains largely impervious to the effects of perspiration. Pang et al. have proposed a skin patch that is both highly breathable and highly draining, and which can be reused. It was inspired by the microchannel network found in tree frog toe pads and the convex cups of octopus suction cups. The patch incorporates octopus-like convex cups on the top surface of its hexagonal structure, which facilitates perspiration-assisted negative-pressure suction cups through a hexagonal prismatic structure. This can enhance adhesion to the skin under conditions of sweating or even running water. Additionally, it can be integrated with diagnostic sensing elements for long-term monitoring of vital signals.^32^

Having a high adhesion capability is the primary requirement for flexible electronic devices applied for continuous detection, and since flexible electronic devices are laminated to the skin surface, this places a high demand on their stretchability. Dong et al. developed a bilayer composite hydrogel with high stretchability, toughness, and adhesion using an *in situ* polymerization process, drawing inspiration from the skin.^164^ The hydrogel is firmly attached to a wide range of surfaces (Figure 12c), establishing a foundation for applications. The hydrogel can be used to detect movement on the skin (Figure 12d) and as a wound healing drug delivery system (Figure 12e). This double-layer composite hydrogel with electromechanical and biocompatible features has great potential for human-computer interaction, health diagnosis and wound treatment. Subsequently, Feng et al. proposed a flexible and ultrathin self-sustaining bioelectronic patch (Biopatch) that can self-attach to the damaged area of an organ and generate bionic electrical signals synchronized with the vagus nerve envelope *in situ* for biomimetic electrical stimulation to adequately meet the personalized needs of tissue regeneration.^165^ A comparison of cardiac signals measured by electrophysiological monitors, piezoelectric film, and patch, respectively, showed that the patch could sensitively detect 6 heartbeats per second in mice with high stability and high sensitivity (Figure 12f,g).

The present flexible electronic devices, which are based on bionics, are designed to achieve higher sensitivity. However, they are unable to take into account aspects such as sweat drainage and long-term application. Consequently, the multifunctional integration of flexible electronic devices at a high level of integration continues to present a significant challenge. Yu et al. were motivated by the occurrence of spontaneous liquid transport on the unique surfaces of natural organisms (e.g., hogweed, cactus, spider silk). They conceptualized a structure capable of facilitating spontaneous and directional liquid transport in three dimensions, which they designated as a three-dimensional liquid diode (3D LD).^166^ This patch can be directly integrated with high-performance flexible circuits through conventional processing to prepare wearable electronic devices that combine high breathability and integration. After applying a commercial electrode patch, a commonly used PDMS substrate and a 3D LD patch on the same user for 20 min of exercise, the results show that the signal-to-noise ratios of the commercial electrode patch and the PDMS electrodes continue to deteriorate with the duration of exercise/sweat, and that reading becomes increasingly difficult as more sweat accumulates on the electrode-skin interface. In contrast, 3D LD-based electrodes with good moisture permeability can provide stable skin-electrode impedance and ECG signals before and after sweating (Figure 12h,i). Moreover, the patch can be continuously detected for about a week. This study promotes the development of breathable electronics from concept to practical application, which helps to improve the user experience in the field of wearable electronics and has broad application potential.

## 5 Conclusions and Outlook

To meet the critical requirement of strong and controllable attachment for medical biointerfaces, various bioadhesive systems found in nature have been studied, and their underlying mechanisms have been elucidated. This review categorizes these bioadhesive systems, ranging from dry to wet to underwater environments, with examples including geckos, tree frogs, and octopuses. The basic principles of interfacial interactions are discussed, based on mechanisms such as van der Waals force, capillary force, negative pressure suction, mechanical interlocking, and chemical adhesion. The underlying unique interfacial liquid and stress-adjusting mechanism have been concluded, where surface micronano structures and special material properties are coupling utilized to regulate the interfacial air/liquid movement and contact stress distribution. For instance, on tree frogs, its two-level concave micropillar surfaces exploit the liquid film self-splitting and self-sucking effects, exhibiting strong boundary friction properties. The miniaturized suction cups inspired by octopuses utilize elasticity-enhanced hydrodynamics to generate “self-sealing” and high suction at the contact interface, converting water into “glue”. Nevertheless, more natural bioadhesive systems remain to be discovered and revealed, particularly those with unique structural features and interfacial dynamic behaviors at the nanoscale, where both nanocharacterization and fabrication present significant challenges. Novel *in situ* interfacial characterization methods based on light spectrum analysis and atomic force microscopy (AFM) can be developed. As more dynamic behaviors of liquid movement, structural deformation, and interfacial stress regulation at the nanoscale are uncovered, a comprehensive theory of bioadhesion can be established, encompassing conditions from dry to wet environments and spanning the micro- to nanoscale, and even the molecular scale.

This review further introduces the varied fabrication methods for bioinspired attachment surfaces and summarizes their applications in medical and healthcare fields. The importance of biomimetic adhesion structures and materials has been declared in improving soft tissue/medical device interfacial adhesion. As medical devices increasingly evolve toward minimally invasive surgical tools and wearable electronics, maintaining adhesion performance has become a critical challenge in developing reliable soft tissue/medical device contact interfaces. Especially under the influence of time-varying biointerfacial factors, e.g. humidity, temperature fluctuations, and tissue secretions or bodily fluids, the attachment of biointerface should be functional and stable in a wide range of environments. Typically, excessive humidity can increase the liquid volume on the adhesive surface, reducing the adhesion strength, while extreme temperature fluctuations may compromise the structural stability of adhesive materials, leading to performance degradation over time. Therefore, improving the stability and reliability of adhesive systems for long-term use, particularly in dynamic and challenging medical environments is important in future research. To mitigate the impact of environmental factors on adhesion durability, materials with moisture resistance, chemical resistance, self-cleaning/self-healing properties can be developed. Adhesive systems integrated with adhesion strength monitoring function and capable of automatic activated self-repair adhesive can also be designed. With these additional functions, bioinspired adhesives are expected to be applied in more diverse applications and facilitate broader applications and breakthroughs in medical and healthcare fields.

## Vocabulary

Interfacial dynamic behaviors: dynamic interactions at contact interfaces during adhesion and friction, including solid–solid interactions, solid–liquid interactions, and solid–liquid–solid interactions
boundary friction: the highest friction during successive wet friction, occurring when the thickness of interfacial liquid film reduces to the nanometer scale
cavitation: the formation of vapor bubbles in a liquid when the local pressure falls below the vapor pressure of the liquid
liquid cavitation threshold: the critical pressure or force at which cavitation occurs in a liquid
cohesive forces of water: the intermolecular forces that cause water molecules to be attracted to each other
